# MYB-bHLH-TTG1 in a Multi-tiered Pathway Regulates *Arabidopsis* Seed Coat Mucilage Biosynthesis Genes Including *PECTIN METHYLESTERASE INHIBITOR14* Required for Homogalacturonan Demethylesterification

**DOI:** 10.1093/pcp/pcad065

**Published:** 2023-06-24

**Authors:** Patrick J Allen, Ross S Napoli, Roger W Parish, Song Feng Li

**Affiliations:** Department of Animal, Plant and Soil Sciences, AgriBio, Centre for AgriBiosciences, School of Agriculture, Biomedicine and Environment, La Trobe University, Bundoora, Melbourne, Victoria 3086, Australia; Department of Animal, Plant and Soil Sciences, AgriBio, Centre for AgriBiosciences, School of Agriculture, Biomedicine and Environment, La Trobe University, Bundoora, Melbourne, Victoria 3086, Australia; Department of Animal, Plant and Soil Sciences, AgriBio, Centre for AgriBiosciences, School of Agriculture, Biomedicine and Environment, La Trobe University, Bundoora, Melbourne, Victoria 3086, Australia; Department of Animal, Plant and Soil Sciences, AgriBio, Centre for AgriBiosciences, School of Agriculture, Biomedicine and Environment, La Trobe University, Bundoora, Melbourne, Victoria 3086, Australia

**Keywords:** *Arabidopsis thaliana*, Cell walls, MBW, Mucilage, Pectin, PMEI, Seed coat, Transcription factors, TTG1

## Abstract

MYB-bHLH-TTG1 (MBW) transcription factor (TF) complexes regulate *Arabidopsis* seed coat biosynthesis pathways via a multi-tiered regulatory mechanism. The *MYB* genes include *MYB5, MYB23* and *TRANSPARENT TESTA2* (*TT2*), which regulate *GLABRA2* (*GL2*), *HOMEODOMAIN GLABROUS2* (*HDG2*) and *TRANSPARENT TESTA GLABRA2* (*TTG2*). Here, we examine the role of *PECTIN METHYLESTERASE INHIBITOR14 (PMEI14)* in seed coat mucilage pectin methylesterification and provide evidence in support of multi-tiered regulation of seed coat mucilage biosynthesis genes including *PMEI14*. The *PMEI14* promoter was active in the seed coat and developing embryo. A *pmei14* mutant exhibited stronger attachment of the outer layer of seed coat mucilage, increased mucilage homogalacturonan demethylesterification and reduced seed coat radial cell wall thickness, results consistent with decreased PMEI activity giving rise to increased PME activity. Reduced mucilage release from the seeds of *myb5, myb23, tt2* and *gl2, hdg2, ttg2* triple mutants indicated that *HDG2* and *MYB23* play minor roles in seed coat mucilage deposition. Chromatin immunoprecipitation analysis found that *MYB5, TT8* and seven mucilage pathway structural genes are directly regulated by MYB5. Expression levels of *GL2, HDG2, TTG2* and nine mucilage biosynthesis genes including *PMEI14* in the combinatorial mutant seeds indicated that these genes are positively regulated by at least two of those six TFs and that TTG1 and TTG2 are major regulators of *PMEI14* expression. Our results show that MYB-bHLH-TTG1 complexes regulate mucilage biosynthesis genes, including *PMEI14*, both directly and indirectly via a three-tiered mechanism involving *GL2, HDG2* and *TTG2*.

## Introduction

In flowering plants, the seed coat develops from two maternally derived ovule integuments following fertilization and protects the embryo from dehydration, pathogen infection and mechanical damage while also involved in seed dormancy and germination (Leon-Kloosterziel et al. 1994, Debeaujon et al. 2000). The *Arabidopsis* seed coat consists of five cell layers, which commence differentiating 5 d after fertilization (Western et al. 2000, Windsor et al. 2000). The innermost endothelial cell layer (ii1) produces proanthocyanidin (PA) and anthocyanin flavonoid compounds, which accumulate in the central vacuole and impart a dark color to the seed coat during maturation (Debeaujon et al. 2003) with PAs incorporated into tannic cell walls (Demonsais et al. 2020).

The epidermal mucilage secretory cells (MSCs) synthesize pectinaceous mucilage, which coincides with the formation of a volcano-like structure, the columella, in the center of each MSC (Beeckman et al., 2000, Western et al. 2000; Windsor et al. 2000). MSCs undergo cytoplasmic rearrangement as mucilage is deposited into the apoplast at the junction of the radial and tangential cell walls (for reviews, see Haughn and Western 2012, Voiniciuc et al. 2015). Following imbibition, the mucilage is rapidly released from MSCs and forms a halo surrounding the seed consisting of an outer water-soluble layer and an inner mucilage layer. The inner layer may be responsible for the attachment of mucilage to the seed coat (Western et al. 2000, Willats et al. 2001, Macquet et al. 2007).

Seed coat mucilage consists of celluloses, hemicelluloses and pectins including rhamnogalacturonan-I and homogalacturonan (HG) (Western et al. 2000, Windsor et al. 2000, Macquet et al. 2007, Golz et al. 2018). HGs undergo methylesterification during biosynthesis in the Golgi apparatus and then demethylesterification following secretion to the cell wall, a process catalyzed by pectin methylesterases (PMEs) and spatially regulated by their inhibitors (PMEIs) (Jolie et al. 2010). *PMEI6* is known to inhibit HG demethylesterification and is required for *Arabidopsis* seed coat mucilage release (Saez-Aguayo et al. 2013). Another *PMEI*, which may be required for mucilage modification, is *PECTIN METHYLESTERASE INHIBITOR14* (*PMEI14*). *PMEI14* is greatly downregulated in *ttg1-1* seeds (Li et al. 2020), but neither its expression levels nor its role in seed coat development are known. PMEI14 is a member of the large PMEI family, several of which have been reported to inhibit PME activities (Jolie et al. 2010, Saez-Aguayo et al. 2013).

MYB-bHLH-WDR (MBW) complexes regulate seed coat mucilage and tannin biosynthesis. The MYB proteins include MYB5, MYB23 and TRANSPARENT TESTA2 (TT2) (Walker et al. 1999, Gonzalez et al. 2009, Li et al. 2009, Xu et al. 2014), while the bHLH proteins include TT8 and ENHANCER OF GLABRA. TRANSPARENT TESTA GLABRA 1 (TTG1) is a WDR protein (Walker et al. 1999, Francoz et al. 2015, Voiniciuc et al. 2015, Xu et al. 2014, Lloyd et al. 2017, Golz et al. 2018). Three tiers of transcription factors (TFs) have been proposed to regulate seed coat mucilage biosynthesis of which *MYB5, MYB23* and *TT2* are designated as tier 3 genes and *GLABRA2* (*GL2*), *HOMEODOMAIN GLABROUS2* (*HDG2*) and *TTG2* as tier 2 genes (Golz et al. 2018, Li et al. 2020). However, evidence supporting the multi-tiered regulation of several mucilage biosynthesis pathway genes is still lacking. Direct binding of tier 3 TFs to the promoters of mucilage pathway structural genes has not yet been determined. Tier 1 TFs participate primarily in the regulation of metabolic genes, while tier 2 TFs regulate both tier 1 genes and specific metabolic gene groups (Li et al. 2020). Tier 3 TFs can directly regulate tier 2, tier 1 and metabolic genes (Li et al. 2020).

We previously proposed a seed coat mucilage biosynthetic pathway (Li et al. 2020). The single, double and triple mutants of the three tier 3 and three tier 2 genes were used to study the transcriptional regulation of *Arabidopsis* seed coat development (Li et al. 2020). This study examines seed coat mucilage deposition in these combinatorial mutants. This study also assesses the function of PMEI14 in seed coat mucilage pectin demethylesterification within the multi-tiered transcriptional pathway by which mucilage biosynthesis and post-synthesis modifications are regulated.

## Results

### *PMEI14* and *PAE1* are expressed in individual seed coat cell layers

**Fig. 1:**
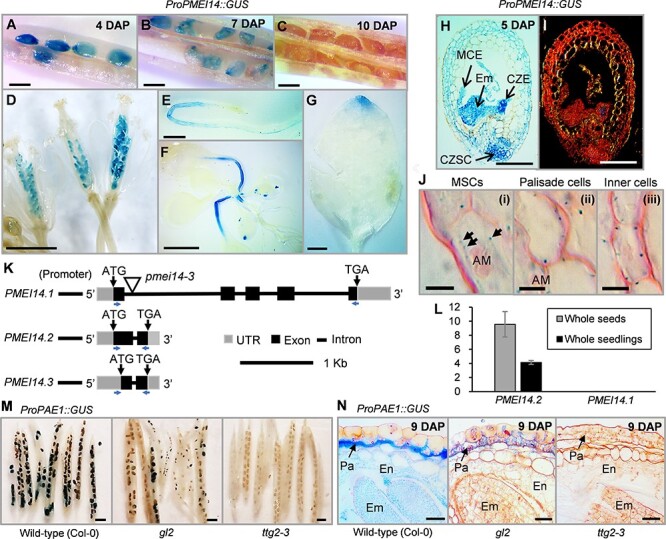
Analysis of *GUS* expression driven by *PMEI14* and *PAE1* promoters and subcellular localization of PMEI14. (A–J) *GUS* expression driven by *PMEI14 (At1g56100)* promoter in developing seeds at (A) 4 DAP, at (B) 7 DAP and (C) no *GUS*, (D) in ovules, (E) in developing roots, (F) in leaf petioles in 2-week-old seedlings and (G) in leaf tips. (H) and (I) A semi-thin section (6 µm) of a *PMEI14* promoter::*GUS* seed at ∼5 DAP stained with safranin (dark-field microscopy with GUS activity in seed coat, embryo and endosperm). (J) Semi-thin sections (3–4 µm) of *PMEI141.2::GFP::GUS* seeds (line 47) at ∼5 DAP counterstained with safranin (arrows indicating GUS activity). (K) Schematic diagram showing the annotated structure of the *Arabidopsis PMEI14* gene based on Expressed Sequence Tag data The Arabidopsis Information Resource (TAIR). The full-length annotation shows five exons separated by four introns [bottom arrows, locations of primers for (L)]. (L) qRT-PCR expression analysis of *PMEI14* mRNA transcriptional splice variants in developing (globular to walking stick stages) wild-type (Col-0) seeds and 2-week-old whole seedlings relative to the *UBIQUITIN10 (UBQ10)* gene *(At4g05320).* (M) *GUS* expression driven by *PAE1 (At1g09550)* promoter in wild-type (Col-0), *gl2* and *ttg2* seeds. Only one silique was chosen from each different PCR confirmed transgenic line (∼10 transgenic lines per mutant background). (N) Cross-sections of *PAE1* promoter::*GUS* expression in developing wild-type (Col-0), *gl2* and *ttg2* seeds. Bars: (A–C) 500 µm, (D, E and M) 1 mm, (F) 5 mm, (G) 2 mm, (H and I) 100 µm, (J) 5 µm and (N) 25 µm. Abbreviations: AM, amyloplasts; CZSC, chalazal seed coat region; CZE: chalazal endosperm, En: endosperm, Em: embryo, MCE: micropylar endosperm, Pa: palisade.

*PMEI14* expression and *PAE1* (for full names and locus details, see **Supplementary Table S2**) expression are strongly downregulated in *ttg1-1* seeds (Li et al. 2020). *PMEI14* and *PAE1* promoter::*GUS* (*β-glucuronidase*) analysis was used to determine the expression patterns of the two genes within each individual seed coat cell layer (**Supplementary Fig. S1**). *PMEI14* promoter–driven GUS activity was detected in seeds at 4 and 7 days after pollination (DAP) but not at 10 DAP (**Fig. 1A****–****C**). In semi-thin sections of seeds (5 DAP), GUS activity was present in the seed coat, endosperm, micropylar endosperm and the young embryo (**Fig. 1H, I**). *GUS* expression was also present in ovules (**Fig. 1D**), developing roots and leaf petioles (**Fig. 1E, F**) and leaf tips (**Fig. 1G**). In 9 DAP seeds, *PAE1* promoter–driven *GUS* expression was strongest in the seed coat palisade cell layer (**Fig. 1M, N**) in wild-type (Col-0), but expression was reduced in *gl2* mutant seeds and absent from *ttg2* mutant seeds (**Fig. 1M, N**), suggesting regulation of *PAE1* expression by GL2 and TTG2.

The three *PMEI14* mRNA splice variants are denoted *PMEI14.1*, *PMEI14.2* and *PMEI14.3* (**Fig. 1K**) (https://www.arabidopsis.org). *PMEI14.1* (699 bp) possesses five exons, while *PMEI14.2* (386 bp) and *PMEI14.3* (309 bp) possess two exons (**Fig. 1K**). Consequently, parts of the first exon sequences of the two shorter transcripts are identical to sequences in the longer transcript (**Fig. 1K**; **Supplementary Fig. S2**). The longer *PMEI14.1* mRNA was not detectable in developing seeds or whole seedlings (**Fig. 1L**). However, using qRT-PCR, the *PMEI14.2* alternative mRNA splice variant was detectable in seeds and seedlings (**Fig. 1L**). Hence, one or both short mRNA splice variants (*PMEI14.2* and/or *PMEI14.3*) are expressed during seed development, while *PMEI14.1* is not expressed. *PMEI14.2* expression was not detected in *pmei14-3* (SM_3_38019) T-DNA insertion mutant seeds, indicating that *pmei14-3* is a null mutant (**Supplementary Fig. S2; Supplementary Table S1**).

To determine the subcellular localization of PMEI14.2, the expression pattern of a *PMEI14.2::GFP::GUS* fusion complementation construct (**Supplementary Fig. S1**) was examined. The cross-sections of resin-embedded GUS-stained siliques (line 47) at ∼5 DAP show PMEI14.2::GUS localized in the vicinity of the plasma membrane of seed coat cells (**Fig. 1J**).

### Abnormal radial cell wall and tighter mucilage attachment in *pmei14-3* seeds

**Fig. 2:**
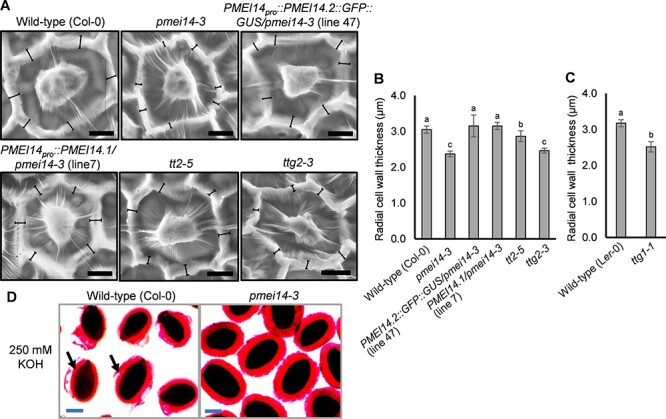
Radial cell wall development and mucilage release in *pmei14-3* mutant seed coats. (A) Scanning electron micrographs of seed coat MSCs of mature wild-type (Col-0), *pmei14-3* (SM_3_38019)*, tt2* and *ttg2* mutants and *PMEI14* complementation lines. Bracketed lines represent regions across radial cell walls that were measured. (B) and (C) Mean radial cell wall thickness values in wild-type (Col-0) and mutant seeds. The values were averaged over 10 biological replicates with at least over 100 cell wall measurements per replicate. Statistical analysis was performed using one-way ANOVA and the Tukey post hoc test. Bars with different letters are significantly different at *P *< 0.05. Data are shown as mean ± SD. (D) Mucilage release of wild-type (Col-0) and *pmei14-3* mutant seeds was observed following treatment with 250 mM KOH. Arrows indicate the released outer water-soluble mucilage layer of wild-type seeds. Scale bars: (A) 10 µm and (D) 200 µm.

PMEIs regulate PME activity to maintain optimal levels of methyl esters in the HG pectin chains of the cell wall (Jolie et al. 2010). Seed coat development in the *pmei14-3* mutant was examined to determine the role of *PMEI14* in seed coat epidermal cell (MSC) wall formation and mucilage modifications. Radial cell wall thickness in MSCs was significantly reduced in *pmei14-3* (2.37 µm, 22% reduction). The cell wall thickness was also examined in *ttg1, tt2* and *ttg2* seeds as the three genes regulate *PMEI14* expression (see later). A reduction in radial cell wall thickness was also observed in these mutants, namely, a mild 6% reduction in *tt2* (2.86 µm), a 19% reduction in *ttg2* (2.46 µm) and a 21% reduction in *ttg1-1* (2.52 µm) compared to wild-type (Col-0 and L*er*-0), respectively (**Fig. 2A****–****C**; **Supplementary Fig. S5, D**). Hence, the TTG1 complexes possessing TT2 may regulate seed coat radial cell wall development via *TTG2* and *PMEI14.* Following alkaline treatment (250 mM KOH), outer water-soluble mucilage from wild-type (Col-0) seeds was partially detached, whereas *pmei14-3* mutant mucilage remained intact (**Fig. 2D**). In EDTA solution, *pmei14-3* seeds exhibited a more punctate distribution of mucilage above the columellae and in the less dense sections of the mucilage (**Supplementary Fig. S3**). The *pmei14-3* mutant was complemented using two *PMEI14* splice variant constructs. As *PMEI14.1* expression is not detectable in seeds, the *PMEI14.1* cDNA sequence was synthesized (GenScript Biotech Corporation, New Jersey) and cloned (**Supplementary Fig. S1, D**). When transformed into *pmei14-3*, the *PMEI14.1* and *PME14.2* cDNA sequences could each restore radial cell wall thickness in *pmei14-3* mutant seeds (**Fig. 2A, B**).

### Altered HG esterification in *pmei14-3* mutant seed coat mucilage and columellae

**Fig. 3:**
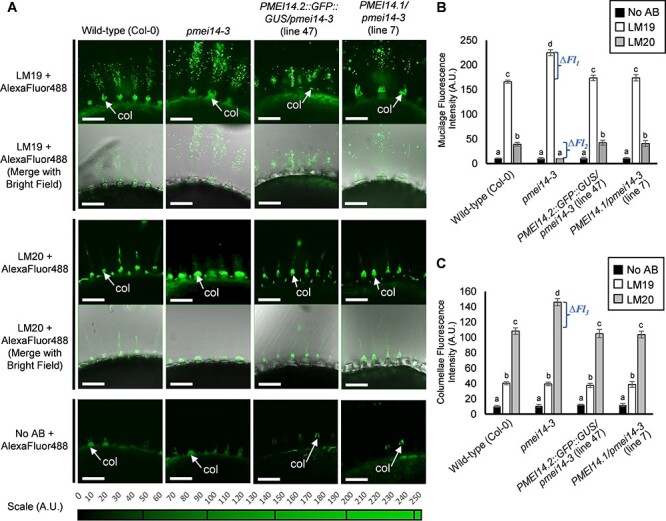
HG pectin methylesterification in wild-type and *pmei14-3* mutant seeds. (A) Immunofluorescence labeling of methylesterfied HG in seed mucilage and columellae of wild-type (Col-0), *pmei14-3* and *PMEI14.2* and *PMEI14.1* complementation lines. Confocal fluoresence microscopy wasperformed and optical sections were obtained showing adherent mucilage released from whole seeds following imbibition. Low or unesterfied HG was labeled with LM19 and highly esterified HG with LM20 in mucilage and columellae. Control replicate experiments were performed without LM19 or LM20. Laser gain values were fixed for each antibody treatment to allow for image comparison and fluorescence quantification relative to the scale of fluorescence intensity. Scale bars: 50 µm. (B) and (C) Quantification of fluorescence intensity following mucilage and columellae immunolabeling of methylesterfied HG in seeds of wild-type (Col-0), *pmei14-3* and *proPMEI14::PMEI14* complementation lines. Fluorescence intensity was determined using maximum fluorescence values with background fluorescence subtracted from each individual image. Immunolabeling was performed on three biological replicates of 20–50 seeds per replicate. Statistical analysis was performed using one-way ANOVA and the Tukey post hoc test. Bars with different letters are significantly different at *P *< 0.05. Data are shown as mean ± SD. Abbreviation: col, columellae.

The degree of HG methylesterification (DM) in wild-type (Col-0) and *pmei14-3* seeds was determined using LM19 and LM20 monoclonal antibodies, which recognize non-esterified and highly esterified HG pectin, respectively (Verhertbruggen et al. 2009). A secondary antibody conjugated to AlexaFluor488 was used to visualize the binding of each primary antibody to HG epitopes using fluorescence microscopy (**Fig. 3A**). Mean maximum fluorescence intensity values from LM19 and LM20 antibody treatments were quantified (**Fig. 3B, C**).

LM19 antibody binding to *pmei14-3* mucilage was increased by 35% (∆Fl_1_) compared to wild-type (Col-0) but was unaffected in columellae (**Fig. 3A****–****C**). In contrast, LM20 antibody binding to *pmei14-3* mucilage decreased by 74.5% (*∆*Fl_2_) compared to wild-type (Col-0), but in columellae, it increased in by 29% (*∆*Fl_3_) (**Fig. 3A****–****C**). These results indicate that HG demethylesterification is enhanced in *pmei14-3* seed coat mucilage.

In the complemented *pmei14-3* mutant lines, binding to mucilage and columellae by LM19 and LM20 was similar to wild-type (**Fig. 3A****–****C**). The complementation of cell wall thickness and the demethylesterification phenotype of *pmei14-3* suggest that the *PMEI14.1* mRNA transcript encodes a PMEI with a similar function to PMEI14.2 (**Supplementary Figs. S1 and S4**).

### MYB5 directly regulates mucilage pathway genes

**Fig. 4:**
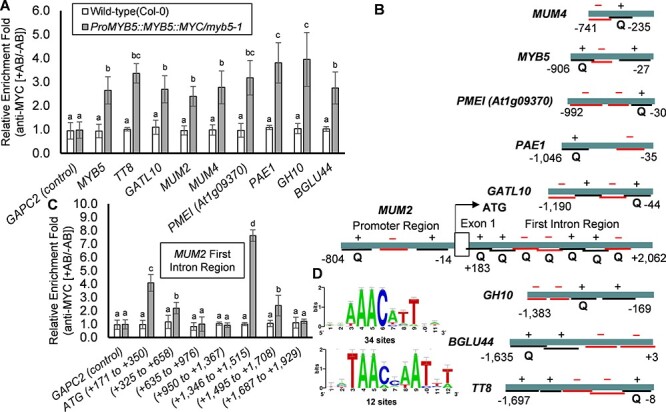
ChIP-qPCR analysis of enriched MYB5-binding regions of TTG1-dependent mucilage pathway genes. (A) ChIP-qPCR analysis. The values represent mean fold enrichment (+AB/−AB, *n* = 3 biological replicates) following normalization using a control sequence from the *Arabidopsis ACTIN7* gene *(At5g09810)* (Supplementary Figure 9). Chromatin immunoprecipitation enrichment values above the threshold of 2-fold were considered as ‘positively enriched’ post-normalization. A sequence from the *GAPC2* gene (*At1g13440*) was used as a non-enriched negative control. Statistical analysis was performed using one-way ANOVA and the Tukey post hoc test. Bars with different letters are significantly different at *P *< 0.05. Data are shown as mean ± SD. (B) A subset of 11 promoters are presented. Underlines and ‘+’ represent enriched regions, while underlines and “−” represent regions tested that were not enriched. Q: Quantified amplicon. (C) Enrichment levels of the first intron of the *MUM2* gene where MYB5 binds to the intron region in at least two locations. Bars represent ±SD. (D) Logos of sequences enriched in promoter and intergenic regions in MYB5 ChIP analysis. Logo bars: ±SE. Abbreviation: AB, anti-MYC antibody; Q, quantified amplicon; SE, standard error.

Chromatin immunoprecipitation (ChIP)-quantitative Polym-erase Chain Reaction (qPCR) analysis was used to determine whether MYB5 directly regulates nine mucilage pathway genes including *PMEI14*. The promoter sequences (∼1000 nucleotides upstream from the ATG start codons) of 12 TTG1-regulated genes (Li et al. 2020) were analyzed using the *cis-*PLACE (www.dna.affrc.go.jp/PLACE) and Arabidopsis Gene Regulatory Information Server (AGRIS) databases (https://agris-knowledgebase.org/) to identify the locations of putative MYB1AT, MYB2AT, MYB1LEPR (MYB G-BOX), MYBATRD22, MYBCORE, MYBCOREEATCYCB1, MYBGAHV, MYBPLANT, MYBPZM, MYB26PS, MYBCONSENSUSAT and MYBST1 binding *cis-*elements upstream of TATA box transcriptional start sites (**Supplementary Table S2**). Enrichment values for each promoter region were quantified using ChIP-qPCR (**Fig. 4A**). From the 12 genes selected for ChIP analysis, 22 out of 48 tested amplicon regions (46%) in 10 promoters were enriched (**Fig. 4B**, **Supplementary Tables S3 and S4**). The nine mucilage pathway genes (**Supplementary Fig. S9; Supplementary Table S4**), namely, *PMEI14*, *PMEI (At1g09370)*, *PAE1*, *GH10*, *BGLU44*, *GATL10*, *MUM2* and *MUM4* promoter regions were all enriched in ChIP assays using a monoclonal antibody directed against the MYB5::MYC fusion protein. The *MUM2* first intron regions plus the *MYB5* and *TT8* promoter regions were also enriched in the assays (**Fig. 4**). Comparative analysis of enriched promoter regions identified (T/A)AAC(A/C/T)N(T/A)(T/A) as a MYB5-binding consensus sequence (**Fig. 4D**; **Supplementary Fig. S8**), consistent with previous studies (Li et al. 2020). The *GATL5* promoter was not significantly enriched in our ChIP experiments. The *PMEI14* promoter was enriched below the 2-fold threshold, while the *TRANSMEMBRANE PROTEIN (At5g60630)* promoter was not enriched (**Supplementary Fig. S9**). These results indicate that the MYB5 protein binds to these promoter regions and suggest that MYB5 may autoregulate its own expression.

### Expression of MYB5 target genes in tier 3 and tier 2 TF mutant seeds

**Fig. 5:**
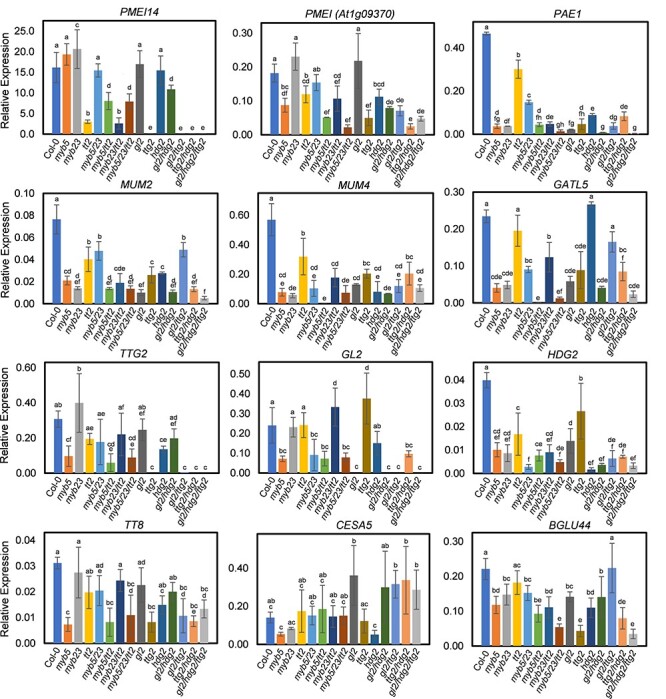
Expression of TTG1-dependent mucilage pathway genes in *myb5*, *myb23*, *tt2* and *gl2*, *hdg2*, *ttg2* combinatorial mutant seeds. qRT-PCR expression analysis of MYB5 target genes in developing seeds (globular to walking stick stages) of single, double and triple mutants. The transcript levels were presented as transcript abundance. Values shown in wild-type (Col-0) and mutant seeds were averaged over three biological replicates. The *UBIQUITIN10* (*UBQ10*) gene (*At4g05320*) was used as an internal reference gene for all experiments. Statistical analysis was performed using one-way ANOVA and the Tukey post hoc test. Bars with different letters are significantly different at *P *< 0.05. Data are shown as mean ± SD.

Expression of MYB5 target genes was investigated using qRT-PCR in single, double and triple mutant seeds of tier 3 mutants (*myb5, myb23* and *tt2*) and seeds of tier 2 mutants (*gl2, ttg2* and *hdg2*). *PMEI14* transcript levels were greatly reduced in *tt2* and *ttg2* mutant seeds, indicating that TT2 and TTG2 are required for *PMEI14* expression (**Fig. 5****Supplementary Fig. S10**). *PMEI (At1g09370)* expression was downregulated in the mutants containing *myb5, tt2, hdg2* and *ttg2* mutations (**Fig. 5**; **Supplementary Fig. S10**). *MUM2, MUM4* and *PAE1* were downregulated considerably in all mutant combinations examined, while *BGLU44* expression was slightly reduced (between 1- and 3-fold), indicating that these genes are positively regulated by the six TFs (**Fig. 5**; **Supplementary Fig. S10**). *CELLULOSE SYNTHASE5 (CESA5)* is required for cellulose biosynthesis in seed coat mucilage and is positively regulated by HDG2 (Sullivan et al. 2011, Kong et al. 2021). *CESA5* expression was downregulated in *myb5, myb23* and *hdg2* single mutants and upregulated in the *gl2* single mutant (**Fig. 5**;). The TF gene *HDG2* was significantly downregulated (3- to 5-fold) in the *myb5, myb23, tt2* and *gl2* mutant combinations. *TTG2* was downregulated in *myb5, tt2* and *hdg2* mutant combinations. *GL2* was downregulated in *myb5* and *hdg2* mutants (**Fig. 5**). The *TT8* tier 3 TF gene was downregulated in *myb5* and *ttg2* mutants (**Fig. 5**). These results show that tier 2 TF genes and mucilage biosynthesis genes including *PMEI14* exhibited significant changes in their expression levels in tier 3 TF mutant seeds.

### HDG2 and MYB23 regulate seed coat mucilage release

**Fig. 6:**
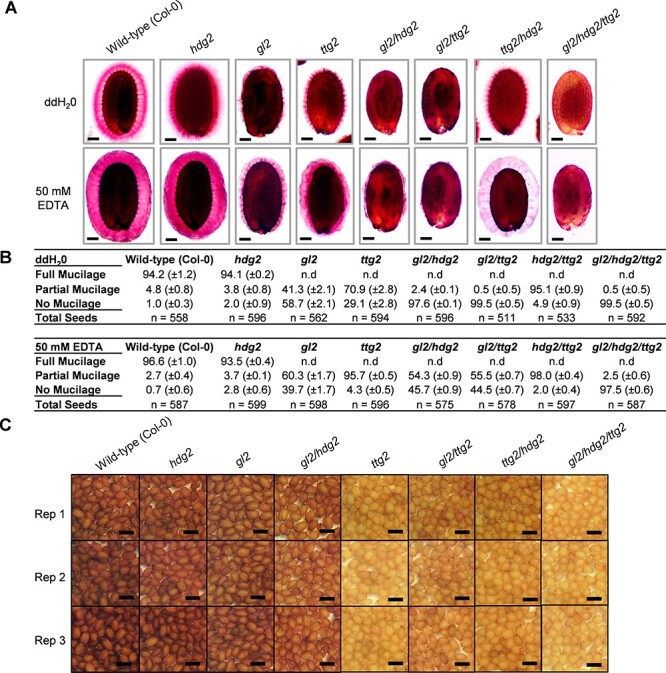
Mucilage release and seed color phenotypes of *gl2, ttg2* and *hdg2* mutant combinations. (A) Levels of mucilage release following staining with 0.05% ruthenium red solution. Dry seeds of wild-type (Col-0) and mutants were shaken in ddH_2_O or 50 mM EDTA treatments for 30 min before staining. (B) Quantification of mucilage release levels in three classes, namely, full, partial or no mucilage release. The data were calculated from three independent experiments and are shown as percentages ± SD. The total values show the total number of seeds examined. n.d., not detected. (C) Seed color phenotypes of wild-type (Col-0) and the mutant combinations in three biological replicates. Scale bars: (A) 100 µm and (C) 500 µm. Abbreviation: n.d., not detected.

The single, double and triple mutants of *MYB5, MYB23, TT2* and *GL2, HDG2, TTG2* were previously used to study the regulation of seed coat biosynthesis pathways (Li et al. 2020). To investigate redundancy between the tier 3 genes and between the tier 2 genes in the regulation of mucilage synthesis, the seeds of the various mutant combinations were stained with 0.05% ruthenium red (**Fig. 6A**). Prior to staining, seeds were shaken in distilled water or 50 mM EDTA solution. The mucilage layer of the *hdg2* single mutant in water (ddH_2_O) was less defined at the surface and thinner compared to the wild-type (**Fig. 6A**). The *gl2 hdg2* double mutant exhibited reduced mucilage release, a more severe mucilage extrusion phenotype than that of either *hdg2* or *gl2* single mutant seeds (**Fig. 6A**). Furthermore, *ttg2 hdg2* mutant mucilage appeared less dense and bound less ruthenium red stain than *ttg2* mucilage following EDTA treatment, a phenotype not present in any other tier 2 mutant (**Fig. 6A, B**). The *ttg2* mutant also exhibited uneven mucilage staining with less dense punctations following EDTA treatment (**Fig. 6A, B**). Thus, the *ttg2* mutants displayed some similarity in mucilage staining pattern to that of the *pmei14* mutant, consistent with *PMEI14* being regulated by *TTG2* (**Fig. 5**). The *gl2 hdg2 ttg2* triple mutant was devoid of mucilage in both treatments, a phenotype more severe than that of the various single or double mutants. These results indicate a novel role for HDG2 in regulating seed coat mucilage synthesis.

The release of seed coat mucilage by the *myb5, myb23* and *tt2* mutants was examined (**Supplementary Fig. 7A and B**). The amount of mucilage released from the seeds of single *myb23* and *tt2* mutants resembled wild-type. The *myb5 myb23* double mutant and the *myb5 myb23 tt2* triple mutant displayed reduced mucilage release when compared to the *myb5* single mutant. Hence, *MYB23* appears to play a minor role in mucilage release (**Supplementary Fig. S7A and B**).

Seed color was examined in the tier 2 and tier 3 TF mutants. The color of *hgd2* seeds was similar to wild-type. The *gl2* single mutant and *gl2 hgd2* double mutant seeds were slightly lighter in color compared to wild-type (Col-0) and the *hdg2* single mutant, implying a minor a minor role in PA biosynthesis (**Fig. 6C**). The *myb5 tt2* and *myb5 myb23 tt2* seeds were slightly lighter in color than *tt2* seeds, indicating that MYB5 is a minor regulator of seed coat color (**Supplementary Fig. S7**).

**Fig. 7:**
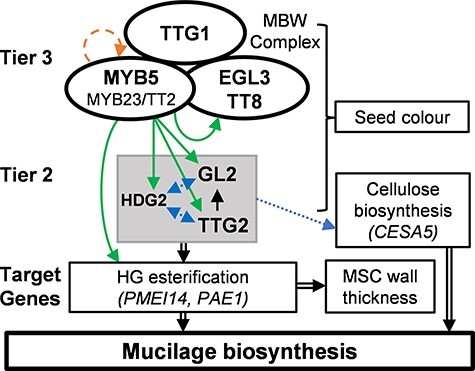
Model of the MYB-bHLH-TTG1-regulated seed coat mucilage pathway. MYB5-bHLH-TTG1 complexes regulate the expression of mucilage biosynthesis genes directly and indirectly via a multi-tiered regulatory pathway comprising transcriptional activators and repressors. The MBW complexes directly regulate mucilage biosynthesis genes and the tier 2 regulator genes: *GL2, HDG2* and *TTG2*. The three tier 2 genes themselves also regulate mucilage biosynthesis genes. Solid arrows indicate modes of gene regulation supported by a combination of transcriptomic, qRT-PCR and ChIP analyses (Li et al. 2020 and this study). Dotted arrows (middle) indicate modes of gene regulation supported by qRT-PCR analysis only. HDG2 was shown to directly regulate *CESA5*, which plays a role in seed coat cellulose biosynthesis (Kong et al. 2021). The dashed arrow (top) indicates direct *MYB5* gene regulation supported by ChIP analysis. The thick compound arrows (bottom) from metabolic genes towards mucilage biosynthesis represent indirect regulation. TTG2 directly regulates *GL2* (Xu et al. 2022).

## Discussion

MBW TF complexes regulate seed coat, trichome and root hair development ( Li et al. 1996, Johnson et al. 2002, Gonzalez et al. 2009, Li et al. 2009, Xu et al. 2014, Li et al. 2020). MBW complexes were previously proposed to regulate *Arabidopsis* seed coat metabolism via a three-tiered regulatory mechanism (Golz et al. 2018, Li et al. 2020). Here, we show that MBW complexes directly and indirectly regulate seed coat mucilage biosynthesis genes including *PMEI14* via a three-tiered mechanism and that *PMEI14* plays a role in seed coat pectin demethylesterification required for cell wall development within the MBW-regulated pathway.

### PMEI14 inhibits HG demethylesterification and is involved in cell wall development

HG pectin demethylesterification plays a role in mucilage maturation (for reviews, see Western 2006, Mohnen 2008). PMEIs act to regulate PME activity, which removes methyl esters from HG pectin chains (Pelloux et al. 2007). The regulation and expression pattern of the TTG1-regulated *PMEI14* gene was examined in this study. The *pmei14-3* mutant exhibited increased HG demethylesterification in seed coat mucilage, suggesting that PME activity is increased as a consequence of decreased PMEI activity. The increased mucilage HG demethylesterification may be responsible for the tighter attachment of the outer layer of mucilage observed in *pmei14-3* seeds via increased levels of Ca^2+^ cross-linked HG (Shi et al. 2018). However, levels of highly esterified HG pectin were increased in the mutant columellae, which may have resulted from the excess of O-methyl compounds, a consequence of HG demethylesterification in mucilage. These results suggest that PMEI14 functions as a PME inhibitor during mucilage synthesis. *PMEI14* expression is also regulated by the MYB52 and ERF4 TFs (Shi et al. 2018, Ding et al. 2021). The MYB5-regulated *PMEI (At1g09370)* (see later) may also participate in the regulation of mucilage demethylesterification. *PMEI6* is the only other gene known to inhibit HG demethylesterification of *Arabidopsis* seed coat mucilage (Saez-Aguayo et al. 2013), while activation of PME activity may occur through proteases such as SUBTILISIN-LIKE SERINE PROTEASE1.7 (SBT1.7)/ARA12 (Rautengarten et al. 2008).

A significant reduction of MSC cell wall thickness occurred in *ttg1, ttg2* and *pmei14* mutant seeds, suggesting that TTG1 complexes regulate MSC cell wall thickness via TTG2, which, in turn, regulates *PMEI14*. However, it is not clear how PMEI14 regulates cell wall thickness as PME-PMEI14 interactions have not yet been reported. In contrast to *pmei14-3*, seeds of the *gatl5-1* mutant produced a thicker cell wall (**Supplementary Fig. S5**). Analysis of single and double mutants of *gatl5-1* and *mum4-4* showed that *GATL5* plays a role in radial cell wall development and both *GATL5* and *MUM4* are also required for columellae formation (**Supplementary Figs. S5 and S11**).

### Transcription pathway regulating seed coat mucilage biosynthetic genes

Seed coat mucilage biosynthesis is regulated by three tiers of TFs including the tier 3 genes *TTG1, MYB5, MYB23* and *TT2* and tier 2 genes *GL2*, *HGD2* and *TTG2* (Golz et al. 2018, Li et al. 2020, Xu et al. 2022). By examining seed coat mucilage in single, double and triple mutant seeds of *myb5, myb23, tt2* and *gl2, hdg2, ttg2* genes, we show that *HDG2* and *MYB23* play minor roles in seed coat mucilage deposition. *HDG2* is expressed throughout the developing seed coat (Nakamura et al. 2006). MYB23 has been suggested to play a role in mucilage deposition as a chimeric MYB23 repressor driven by the *35S* promoter reduced mucilage deposition in the seed coat (Matsui *et al.*, 2005). We also found that *GL2* plays a minor role in seed coat PA biosynthesis consistent with previous studies (Wang et al. 2015).

The ChIP assay and qRT-PCR were used to determine the transcription pathway regulating seed coat mucilage biosynthesis. While qRT-PCR analysis of TF mutants can determine the expression levels of the TF target genes, the analysis cannot by itself distinguish the direct or indirect modes of regulation of the target genes by the TFs as the downregulation might have resulted from the developmental defects of the seed coat tissues. Consequently, the ChIP assay is required to ascertain the binding of a TF to the target promoters. The ChIP assay targeting direct MYB5-DNA binding identified mucilage pathway genes, namely, *PMEI(At1g09370)*, *PAE1*, *GH10*, *BGLU44*, *GATL10*, *MUM2, MUM4, MYB5* and *TT8*, as direct targets of MYB5. These MYB5 target genes are each co-expressed with *MYB5* during the various stages of seed development (**Supplementary Fig. S12**). MYB5 binding to *MYB5* and *TT8* promoter regions indicated autoregulation of some MBW complex genes. TT8 is also known to autoregulate its expression (Baudry et al. 2006). TTG2 may also regulate *TT8* indirectly as *TT8* expression was reduced in *ttg2-3* mutant seeds (**Fig. 5**). The enriched *MUM4* promoter suggests that MYB5 binds to the *MUM4* 5ʹUTR region, downstream of the small promoter required for *MUM4* expression in seeds (Dean et al. 2017). Hence, MYB5 binding to the *MUM4* 5ʹUTR region may enhance the expression of *MUM4* in seed coats. The first intron regions in *MUM2* were also enriched in the ChIP assay, suggesting that this intron plays a role in regulating *MUM2* expression in seeds. The MYB5 binding consensus sequences (T/A)AAC(A/C/T)N(T/A)(T/A) in the nine enriched promoters and the *MUM2* first intron are similar to previously identified MYB5 DNA-binding sequences (Li et al. 2020). These results indicate that MYB5 directly regulates the seed coat mucilage biosynthetic pathway (**Supplementary Fig. S11**).

The qRT-PCR expression analysis using tier 3 and tier 2 mutants showed that six mucilage pathway genes including *PMEI14* are co-regulated by tier 3 and tier 2 TFs. *PMEI14* expression was greatly reduced in *ttg1* and *ttg2* seeds, while expression was also downregulated in *tt2* seeds. These results suggest that TT2-bHLH-TTG1 complexes, in partial redundancy with MYB5, may directly regulate *PMEI14* expression with additional regulation via TTG2. It remains to be determined whether HG demethylesterification is inhibited in seed coat mucilage of *tt2* mutant seeds to a degree similar to *pmei14-3* mutant seeds. The results of qRT-PCR analysis of *PAE1* are consistent with the *PAE1* promoter analysis, which found expression was strongly downregulated in the *gl2* and *ttg2* single mutants (**Fig. 1**). *CESA5* is positively regulated by MYB5, MYB23 and HDG2 but repressed by GL2 (Tominaga-Wada et al. 2009). *CESA5* was shown to be downregulated in *hdg2* mutant seeds (Kong et al. 2021), while *CESA5* promoter activity was upregulated in *gl2* mutant roots (Tominaga-Wada et al. 2009). *HDG2* was downregulated in *myb5* and *myb23* mutant combinations, and its promoter is bound by MYB5 (Li et al. 2020), indicating that *HDG2* is regulated by tier 3 TFs. *HDG2* is also regulated by GL2 and TTG2, which themselves are regulated by HDG2 (**Figs 5** and **7**). *HDG2* has previously been shown to regulate *GL2* (Kong et al. 2021). Additionally, TTG1 and TTG2 proteins are capable of direct interaction as the two TFs bind in a yeast two-hybrid assay (Pesch et al. 2014). The tier 3 TF *TT8* is positively regulated by MYB5 and TTG2 (**Figs 5** and **7**).

While MBW complexes are master regulators of the multi-tiered network in the seed coat, the Complexes themselves are regulated in a variety of ways. TTG1 activity, for example is regulated by phosphorylation, which prevents its interaction with TT2 (Li et al. 2018), while MYB5 may be regulated by CASEIN KINASE2 BETA3 (CK2β3) as the two proteins interact directly in a yeast two-hybrid system (Napoli et al. 2021).

In summary, we have provided evidence supporting a multi-tiered model of transcriptional regulation of seed coat biosynthesis pathways with particular focus on regulation of MBW-dependent mucilage biosynthesis genes (**Fig. 7**). *PMEI14* plays a role in seed coat pectin demethylesterification and MSC cell wall thickness regulated by tier 3 and tier 2 TFs (**Fig. 7**). MBW complexes directly regulate several mucilage biosynthesis genes and the tier 2 TF genes *GL2, TTG2* and *HDG2* which in turn regulate mucilage biosynthesis and post-synthesis modification genes within the MBW-regulated pathway (**Fig. 7**).

## Methods and Materials

### Plant material and growth conditions

*Arabidopsis thaliana* ecotypes *Columbia* (Col-0) and *Landsberg erecta* (L*er*-0) were used as wild-type controls. The T-DNA insertion mutant lines *gatl5-1* (SALK_106615; Kong et al. 2013), *mum4-4* (SALK_085051C), *pmei14-3* (SM_3_38019), *gl2* (SALK_039825C), *hdg2-3* (SALK_138646C), *ttg2-3* (SALK_148838) and *tt2-5* (SALK_00560) were obtained from the Nottingham Arabidopsis Stock Centre. The *myb5-1, myb23-1* and *ttg1-1* mutant lines used in this study were previously described in Li et al. (2009) and Li et al. (2020). All T-DNA and mutant lines are in the Col-0 background with the exception of *ttg1-1*, which is in the L*er*-0 background (Koorneef 1981, Koornneef et al. 1982, Shirley et al. 1995). The *ProMYB5::MYB5::MYC/myb5-1* lines used in chromatin immunoprecipitation and the *myb5/myb23/tt2* and *gl2/hdg2/ttg2* triple mutants have been previously reported by our laboratory (Li et al. 2020). The *gatl5/mum4* double mutant was created by crossing homozygous lines and identifying homozygous double mutant plants in F_2_ and F_3_ generations by PCR screening and by mucilage release phenotypes. All plants were germinated from seed on Murashige and Skoog medium at 22°C under constant illumination (110 μmol/m^2^/s) and grown on soil at 22°C under 16 h/day of illumination (140 μmol/m^2^/s). T-DNA insertion lines were germinated under the appropriate antibiotic selection.

### Plasmid construction and plant transformation

Promoter::*GFP::GUS* fusion constructs were generated by PCR amplification from Columbia (Col-0) genomic DNA. *PMEI14* (694 bp) promoter and *PAE1* (1,035 bp) promoter regions upstream of the respective ATG start codon were PCR amplified, and the PCR fragments were cloned into the pENTR/D-TOPO entry vector (Invitrogen, Waltham, Massachusetts). Entry clones were sequenced to ensure that no PCR induced errors were present and recombined into the pKGWFS7 binary *GFP::GUS* vector (Karimi et al. 2002) using the GATEWAY cloning strategy (Invitrogen). For *PMEI14* complementation and subcellular localization experiments, a PCR fragment containing a 993-bp *PMEI14* promoter upstream of the ATG and the full-length *PMEI14.2* splice variant (480 bp) was amplified and cloned into the pENTR/D-TOPO entry vector (Invitrogen). Entry clones were sequenced and recombined into the pGWB501 binary vector (Nakagawa et al. 2007) using the GATEWAY cloning strategy (Invitrogen). The resulting constructs were transformed into *Agrobacterium tumefaciens* (GV3101) by electroporation. Transgenic plant lines were generated using the floral drip method adapted from Clough and Bent (1998). The presence of each transgene was verified using PCR. Promoter- and gene-specific primers used for cloning are listed in **Supplementary Table S5**.

### Sectioning of embedded seeds

Whole developing seeds were embedded using the methods described in Western et al. (2000) with some modifications. Siliques were fixed in 50% formaldehyde, 50% acetic acid solution to minimize loss of mucilage from older seeds, vacuum infiltrated for 1 h, embedded in London Resin (LR) White solution and sectioned and stained with 1% (w/v) toluidine blue in 1% (w/v) sodium borate. GUS-stained tissues were counterstained with 1% (w/v) safranin as an alternative to toluidine blue.

### Histochemical analysis of transformed *Arabidopsis* plants

Plant tissue was incubated in X-gluc solution at 37°C for 16 h as described in Jefferson et al. (1987). Chlorophyll was leached from the plant tissue by incubating overnight in 70% ethanol. GUS staining was examined under a dissecting microscope. Developing seeds at specific time points were obtained by tagging flowers on the day of pollination and dissecting siliques at the specified number of days thereafter.

### Scanning electron microscopy

Scanning electron microscopy (SEM) was performed using a Hitachi TM3030 Plus Tabletop Scanning Electron Microscope and manufacturer’s software. Images were generated using an excitation voltage of 15 kV and Backscattered Secondary Electron imaging settings. Quantification of seed coat epidermal radial cell wall thicknesses was performed as described by Walker et al. (2011). Statistical analysis was performed using Microsoft Excel.

### Immunofluorescence of *Arabidopsis* seed mucilage

Two primary monoclonal antibodies were used for immunolabeling of *Arabidopsis* seeds; LM19 and LM20 (PlantProbes, Paul Knox Laboratory, University of Leeds, UK). Mature *Arabidopsis* seeds were imbibed and fixed as described in Macquet et al. (2007). Epitope demasking was performed using an adapted protocol from Leon and Sormunen (1998) by heating samples in 8 mM sodium citrate and 2 mM citric acid in a microwave oven until boiling and then washing three times in phosphate-buffered saline (PBS). Samples were immunolabeled by incubating for 4 h at 37°C with a slow shake in 50 µl of primary antibody diluted 1:10 in PBS containing 1% (w/v) bovine serum albumin (BSA), washed three times in PBS and incubated for 2 h at 37°C with a slow shake in 2 µl of secondary antibody, goat anti-rat-IgG antibody conjugated to Alexa Fluor 488 (Cell Signaling Technology, Danvers, Massachusetts) diluted 1:500 in PBS containing 1% (w/v) BSA. Samples were washed three times in PBS and mounted in SlowFade Gold Antifade reagent (Invitrogen). Cover slips were sealed with clear nail varnish, and samples were analyzed using a fluorescence scanning confocal microscope (Leica TCS SP2). Laser gain values were fixed within a given experiment to facilitate image comparison.

### qRT-PCR analysis

Developing seeds (globular to heart stages) were isolated from young siliques and manually dissected in RNAlater solution (Thermo Fisher, Waltham, Massachusetts). Total RNA was extracted from developing seeds, seed coats and embryos using the RNeasy plant kit (Qiagen, Venlo, Netherlands). First-strand cDNA synthesis was performed according to the manufacturer’s instructions (Invitrogen Superscript III reverse transcriptase and reagents). Expression analysis was performed for each gene compared to *UBIQUITIN10 (At4g05320)* as a stable qRT-PCR reference gene for seed tissue (Czechowski et al. 2005, Li et al. 2020). qRT-PCR was performed using the SensiFAST SYBR and Fluorescein master mix (Meridian Bioscience, Cincinnati, Ohio) on the QuantStudio system (Life Technologies, Waltham, Massachusetts). The PCR conditions are as follows: 95°C for 2 min, 36 cycles at 95°C for 10 s, 57–62ºC for 30 s, 72°C for 30 s, one cycle at 72°C for 5 min. Data were analyzed using the iQ5 (Bio-Rad, Hercules, California) software, and differences in gene expression were calculated using the 2^(−deltaCT)^ analysis method. Gene-specific primers are listed in **Supplementary Table S5**.

### ChIP assay

ChIP experiments were performed as described by Li et al. (2020) using a published protocol (Saleh et al. 2008) with some modifications. Approximately two grams of developing silique tissue was harvested from 5- to 6-week-old *Arabidopsis myb5-1* mutant plants containing the *MYB5::MYC* transgene, immediately frozen in liquid nitrogen and then stored at −80°C until enough material has been collected. An anti-myc antibody (c-MYC sequence, ab9132; Abcam) was used to enrich AtMYB5::MYC protein/DNA complexes. ChIP-PCR was performed using a standard GoTaq (Promega Corporation, Madison, Wisconsin) PCR, 50 µL of total reaction volume and the following cycling parameters: first cycle, 95°C for 4 min; 36 cycles of 95°C for 30 s, 50°C for 30 s and 72°C for 30 s; and one cycle of 72°C for 5 min. ChIP-qPCR was performed on enriched several regions using the SensiFAST SYBR and Fluorescein master mix (Bioline) on the QuantStudio system (Life Technology). The PCR conditions used are described earlier. Data were analyzed using the iQ5 (Bio-Rad) software, and differences in gene expression were calculated using the 2^(−deltaCT)^ analysis method. Motifs were analyzed from each enriched amplicon using the oligo-analysis motif discovery tool from Regulatory Sequence Analysis Tools (http://rsat.eead.csic.es/plants/). Gene-specific primers are listed in **Supplementary Table S5**.

## Supplementary Material

pcad065_Supp

## Data Availability

All the main data supporting the findings in this study are available within the article and its supplementary information. Materials used in this study are available from the corresponding author upon request.

## Accession Numbers

Sequence data from this article can be found in the Arabidopsis Genome Initiative or GenBank/European Molecular Biology Laboratory (EMBL) databases under the following accession numbers: *TTG1 (At5g24520), MYB5 (At3G13540), MYB23 (At5g40330), TT2 (At5g35550), TT8 (At4g09820), GL2 (At1g79840), TTG2 (At2g37260), HDG2 (At1g05230), PMEI14 (At1g56100), PMEI (At1g09370), PAE1 (At1g09550), GATL5 (At1g02720), GATL10 (At3g28340), GH10 (At4g33810), MUM2 (At5g63800), MUM4 (At1g53500), BGLU44 (At3g18080), GAPC2 (At1g13440), ACTIN7 (At5g09810)* and *UBQ10 (At4g05320).* The cloned *PMEI14.1 (At1g56100.1)* nucleotide sequence is available through GenBank/National Centre for Biotechnology Information (NCBI) under number NM_104488.1.
