# Supplementary material for: MYB-bHLH-TTG1 in a Multi-tiered Pathway Regulates Arabidopsis Seed Coat Mucilage Biosynthesis Genes Including PECTIN METHYLESTERASE INHIBITOR14 Required for Homogalacturonan Demethylesterification
Source: Plant Cell Physiol. 2023 Jun 24;64(8):906–19. doi: 10.1093/pcp/pcad065 (PMC10434736; doi:10.1093/pcp/pcad065)
Supplement: pcad065_Supp [file pcad065_supp.zip › suppl_data/pcp-2023-e-00073-File009.pdf]

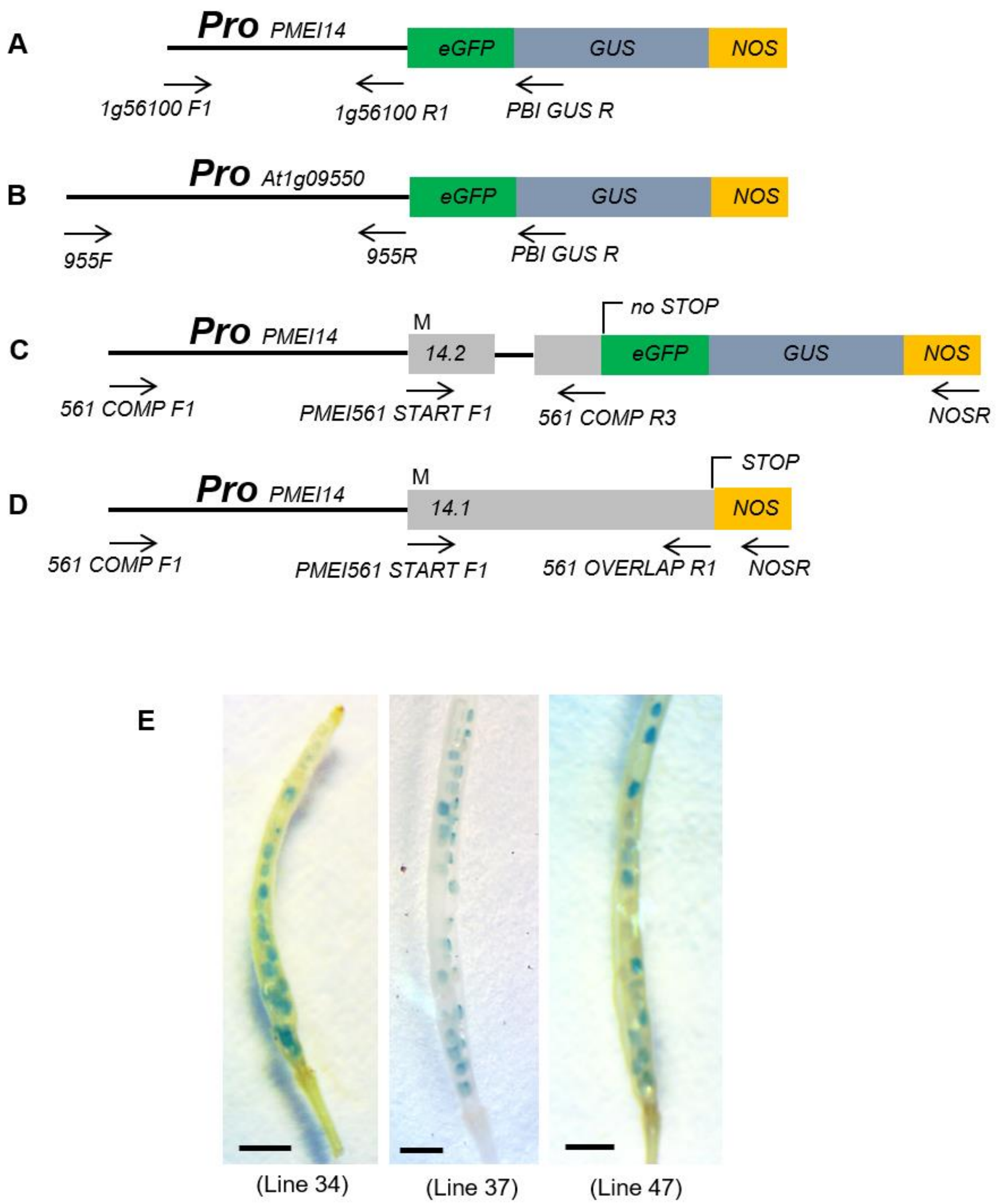

Supplemental Figure 1. Schematic diagrams of *Promoter::Reporter* and *PMEI14* complementation constructs used in this study and histochemical analysis of *ProPMEI14::PMEI14.2::eGFP::GUS* lines.

(A) and **(B)** Schematic representation of the *ProPMEI14::eGFP::GUS* and *ProAt1g09550::eGFP::GUS* constructs. Coloured blocks represent the enhanced *GFP (eGFP)* and *GUS* reporter genes (green and blue, respectively) and a *NOS* terminator (yellow). The solid lines represents the 694 nucleotide *PMEI14* promoter and the 1,035 nucleotide *PAE1 (At1g09550)* promoter, respectively. **(C)** and **(D)** Schematic representations of the *ProPMEI14::PMEI14.2::eGFP::GUS* and *ProPMEI14::PMEI14.1* complementation constructs. Coloured blocks represent the following: *PMEI14.*2 or *PMEI14.1* gene sequences (grey), enhanced *GFP (eGFP)* and *GUS* reporter genes (green and blue, respectively) and a *NOS* terminator (yellow). The solid lines represent the 1,021-nucleotide *PMEI14* promoter. **(E)** Moderate *GUS* expression (blue) in developing seeds at 5 DAP (lines 34, 37 and 47). Scale bars: 1 mm.

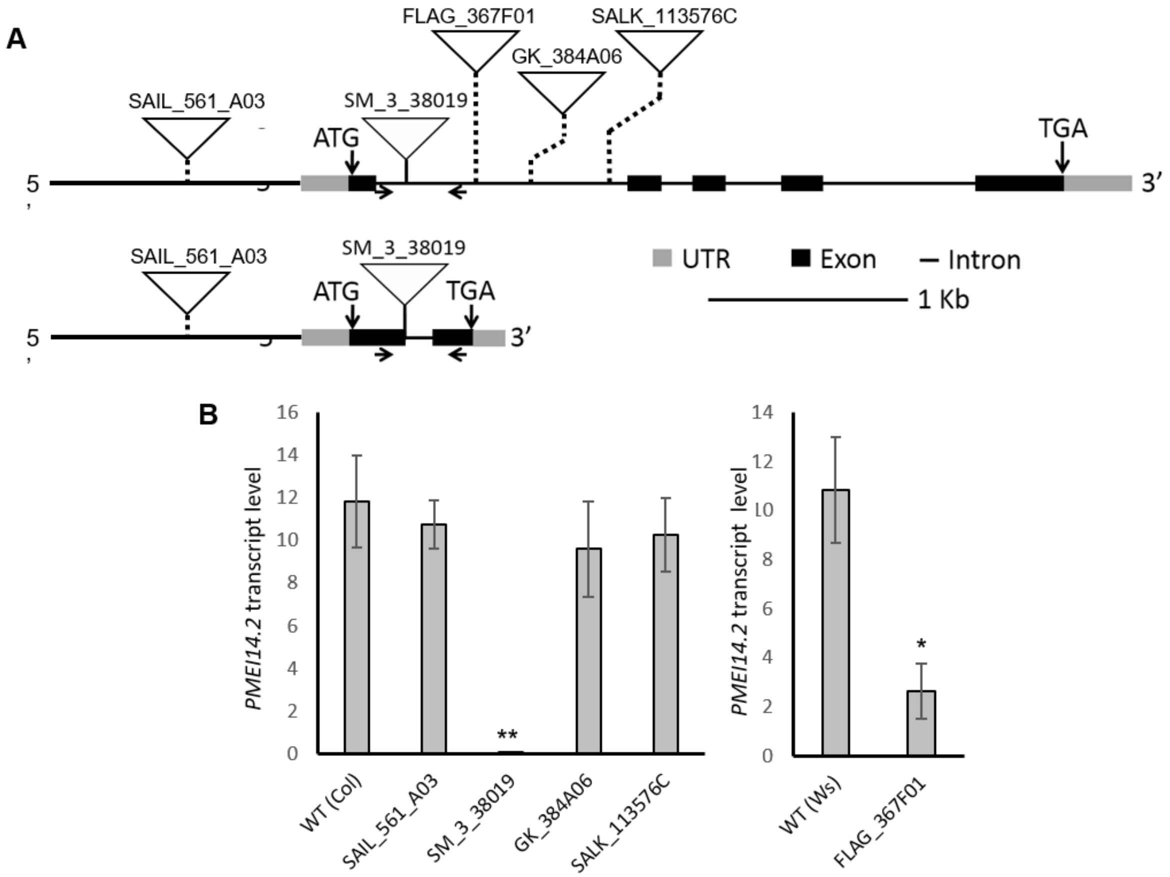

Supplemental Figure 2. Schematic representation and expression analysis of *PMEI14* T-DNA insertion mutants used in this study.

(A) *PMEI14* gene structure. Triangles represent T-DNA insertions. Dotted lines represent T-DNA insertions that are not located within the *PMEI14.2* mRNA coding region. Solid coloured boxes represent untranslated regions (UTR) (grey) and exons (black). Solid lines following the ATG represent introns. The SAIL_561_A03 insertion is in the *PMEI14* promoter region approximately 500 nucleotides upstream of the ATG. The FLAG_367F01, GK_384A06 and SALK_113576C insertions are located within the first intron of the *PMEI14.1* sequence and are approximately 600, 800 and 1,000 nucleotides downstream of the ATG, respectively. SM_3_38019 (*pmei14-3*) is the only T-DNA insertion that is located within the coding region of the *PMEI14.2* annotated sequence. Horizontal arrows underneath the gene annotations represent locations of primer sequences used in qRT-PCR analysis. **(B)** Histogram of *PMEI14.2* expression in insertion mutant lines. *PMEI14.2* expression analysis in developing seeds (globular to walking stick stages) of wild-type (Col-0), SAIL_561A03, SM_3_38019, GK_384A06 and SALK_113567C presented as transcript abundance relative to *UBQ10.* SM_3_38019 is a null mutant. *PMEI14.2* expression analysis in wild-type (Ws) and FLAG_367F01 presented as transcript abundance relative to *UBQ10.* FLAG_367F01 shows reduced *PMEI14.2* expression but is not a null mutant. Values shown were averaged over 3 biological replicates. Statistical difference between wild-type and mutant lines was calculated using Student's T-test (two-tailed, n=3) where *, P<0.05 and **, P<0.01. Error bars represent ±SD.

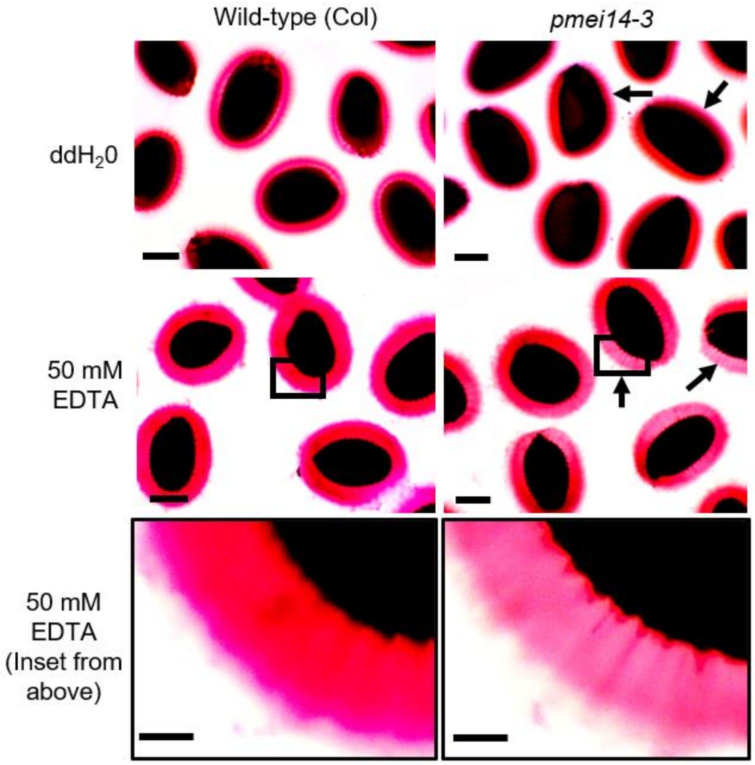

Supplemental Figure 3. Mucilage release phenotypes from wild-type (Col) and *pmei14-3* mutant seeds

Arrows indicate a more punctate distribution of mucilage above the columellae of imbibed *pmei14-3* mutant seeds while the amount of mucilage is not affected when compared to wild-type (Col). Scale bars: 200 µm and 50 µm (inset images).

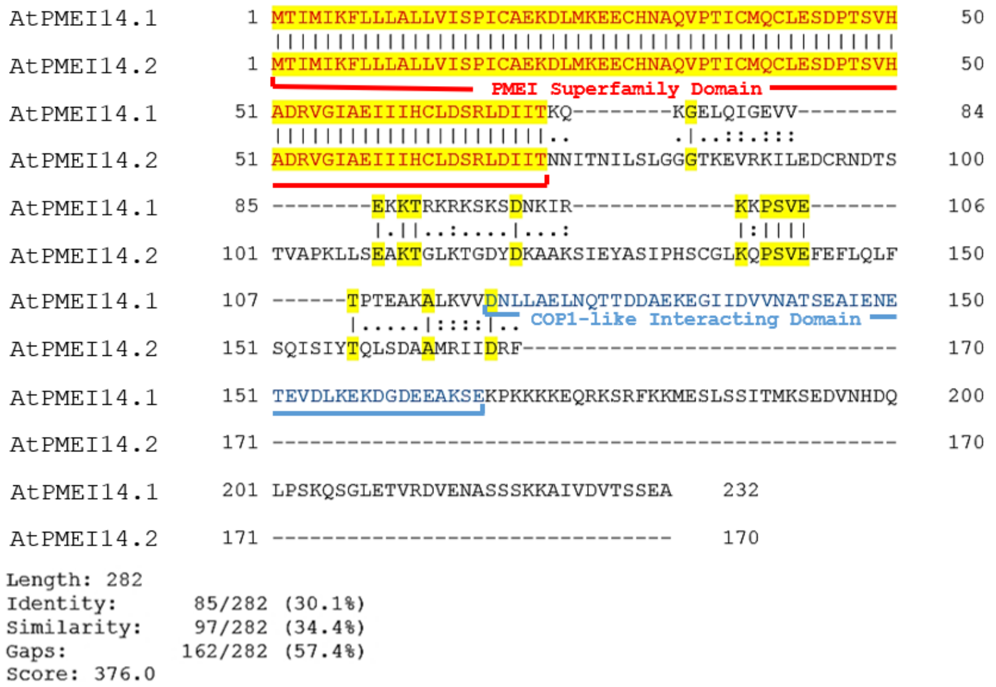

Supplemental Figure 4. PMEI14 amino acid sequence analysis.

Amino acid sequence alignment showing conserved regions in the PMEI14.1 and PMEI14.2/PMEI14.3 proteins. Yellow highlighted regions are conserved between both PMEI14 protein variants. Alignment was generated using the Clustal 2.1 multiple sequence alignment tool (http://www.ebi.ac.uk/Tools/msa/clustalw2/).

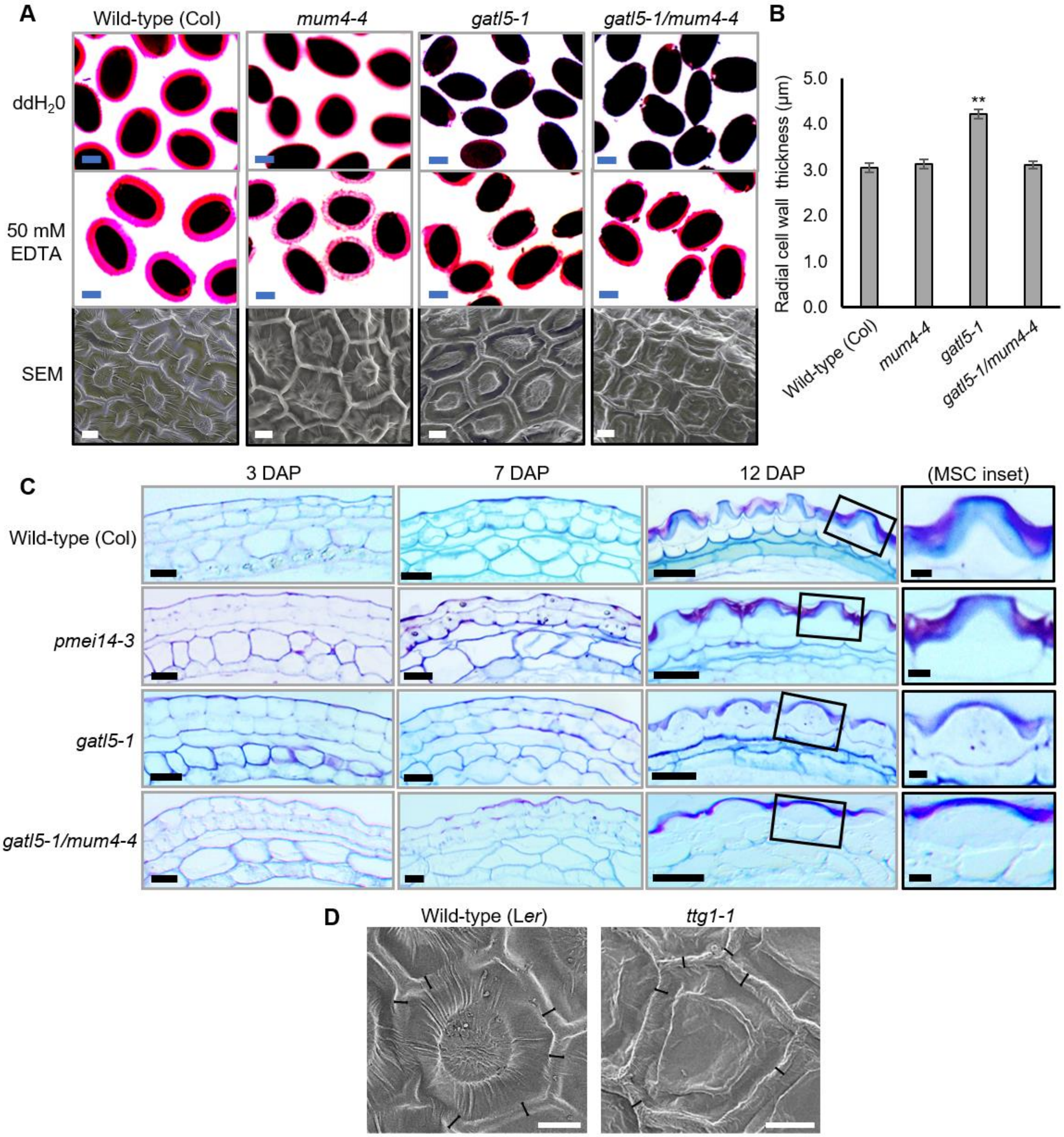

Supplemental Figure 5. Mucilage release and cell wall phenotypic analysis of single and double mutant seeds.

(A) Mature single and double mutant seeds were stained for mucilage release with 0.05% Ruthenium Red after pre-treatment with water or EDTA. Scanning electron micrographs of mature wild-type (Col), *gatl5-1, mum4-4* (SALK_085051C) and *gatl5-1/mum4-4* double mutant seed coat epidermal cell morphology. The *gatl5-1/mum4-4* double mutant seeds display flattened columellae compared to the *gatl5-1* or *mum4-4* single mutants. **(B)** Histogram showing the radial cell wall thickness of the wild-type (Col), *mum4-4, gatl5-1* and *gatl5-1/mum4-4* double mutant seed coat epidermal cells. Mean values are presented from 10 biological replicates (n=10) (at least 100 cell wall measurements per replicate). Statistical analysis was performed using Student's t-test (n=10) where *P < 0.05, **P < 0.01. Error bars represent ±SD. **(C)** Semi-thin sections (4-6 um) of developing wild-type (Col-0) and *pmei14-3, gatl5-1* and *gatl5-1/mum4-4* mutant seeds at 3 DAP, 7 DAP and 12 DAP. The mutant seeds are not morphologically different from wild-type at 3 DAP and 7 DAP. At 12 DAP, the *pmei14-3* mutant epidermal cells and columella appear larger and flatter than wild-type (arrowheads) while *gatl5-1* mutant epidermal cells have less mucilage and enlarged columellae (inset). The *gatl5-1/mum4-4* double mutant displays an additive phenotype. DAP: days after pollination. **(D**) Hexagonal seed coat mucilage secretory cells (MSCs) of *ttg1* mutant seeds display reduced radial cell wall thickness compared to wild-type. Bracketed lines represent regions across radial cell walls that were measured. Representative images are shown. Scale bars: 200 µm (mucilage images), 10 µm (SEM images), 25 µm (section images), 5 µm (inset images).

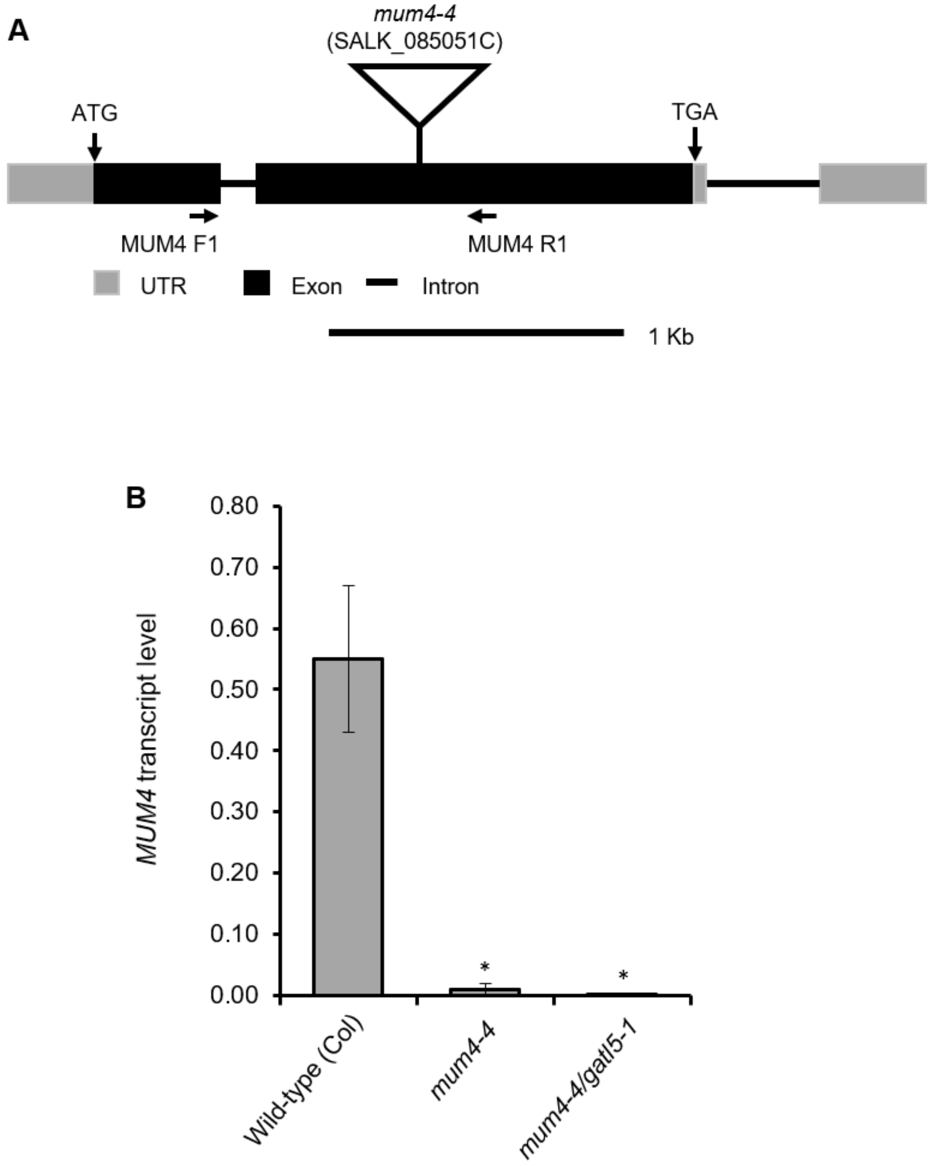

Supplemental Figure 6. T-DNA location and expression analysis of *mum4-4* and *gatl5-1/mum4-4* double mutant seeds.

(A) Schematic representation of the *MUM4* gene showing the location of the *mum4-4* (SALK_085051C) T-DNA insertion within the second exon and screening primers used for the identification of homozygous plants. **(B)** *MUM4* expression analysis wild-type (Col-0), *mum4-4* (SALK_085051C) mutant and *gatl5-1/mum4-4* double mutant developing seeds (globular to walking stick stages) presented as transcript abundance relative to *UBQ10*. Values shown were averaged over 3 biological replicates. Statistical difference between wild-type and mutant lines was calculated using Student's T-test (two-tailed, n=3) where *, $P<0.05$. Error bars represent ±SE.

A

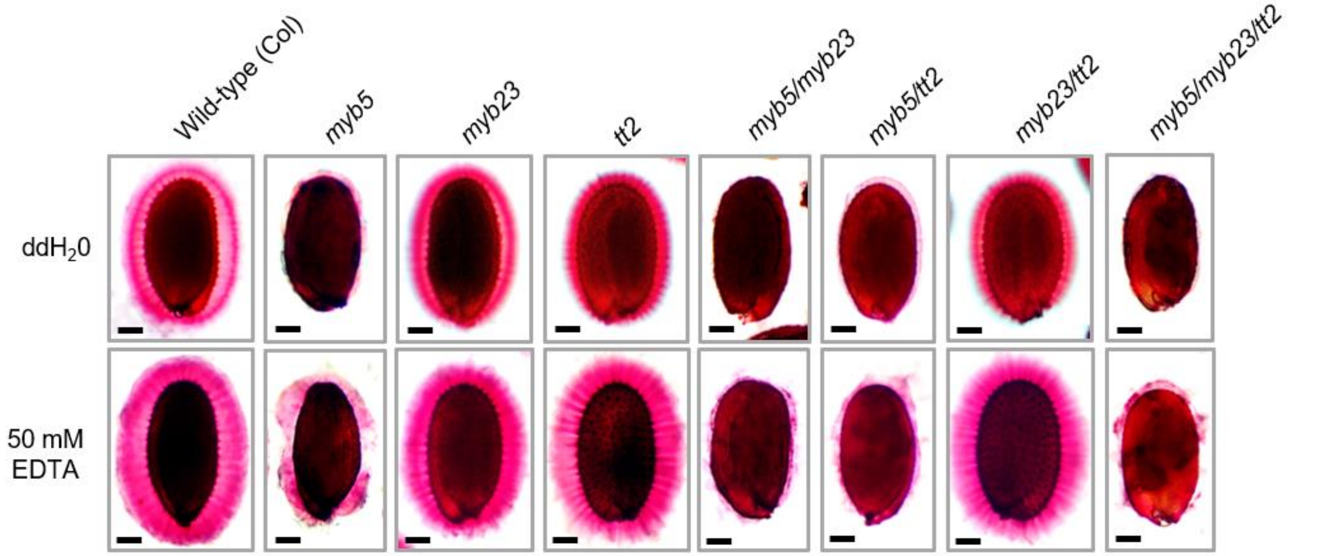

B

ddH$_2$0	Wild-type (Col)	*myb5*	*myb23*	*tt2*	*myb5/myb23*	*myb5/tt2*	*myb23/tt2*	*myb5/myb23/tt2*
Full Mucilage	94.3 (±1.1)	n.d	92.8 (±0.7)	95.3 (±0.4)	0.5 (±0.5)	n.d	99.5 (±0.5)	n.d
Partial Mucilage	4.6 (±0.7)	70.6 (±6.0)	5.7 (±0.4)	3.2 (±0.4)	37.0 (±4.2)	95.2 (±0.3)	0.5 (±0.5)	54.8 (±0.7)
No Mucilage	1.1 (±0.6)	29.4 (±6.0)	1.5 (±0.6)	1.5 (±0.4)	62.5 (±4.4)	4.8 (±0.3)	n.d	45.2 (±0.7)
Total Seeds	n = 597	n = 590	n = 597	n = 590	n = 582	n = 589	n = 583	n = 586

50 mM EDTA	Wild-type (Col)	*myb5*	*myb23*	*tt2*	*myb5/myb23*	*myb5/tt2*	*myb23/tt2*	*myb5/myb23/tt2*
Full Mucilage	96.4 (±0.6)	n.d	94.5 (±1.1)	95.8 (±0.2)	1.2 (±0.5)	n.d	99.8 (±0.3)	n.d
Partial Mucilage	3.0 (±0.4)	91.7 (±1.0)	4.6 (±1.1)	3.6 (±0.4)	76.7 (±1.5)	96.3 (±0.9)	0.2 (±0.3)	83.7 (±1.1)
No Mucilage	0.7 (±0.2)	8.3 (±1.0)	0.9 (±0.4)	0.7 (±0.1)	22.1 (±12)	3.7 (±0.9)	n.d	16.3 (±1.1)
Total Seeds	n = 605	n = 614	n = 579	n = 593	n = 602	n = 613	n = 594	n = 609

C

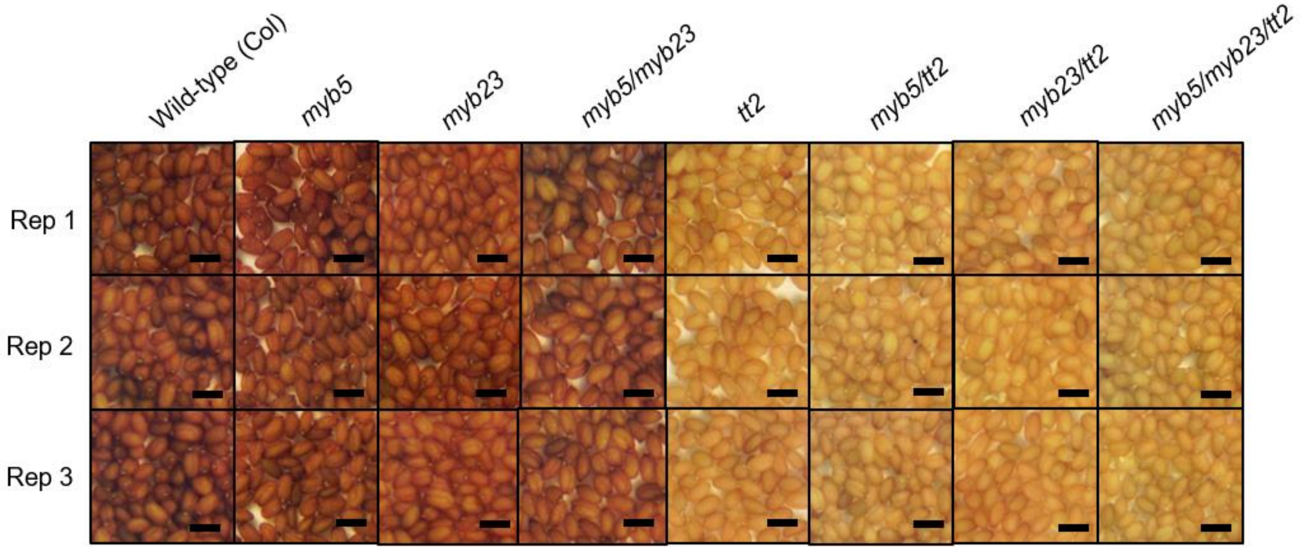

Supplemental Figure 7. Mucilage extrusion and seed colour phenotypes of wild-type (Col) and tier 3 level *myb* mutant combinations.

(**A**) Levels of mucilage extrusion following staining with 0.05% Ruthenium Red solution. Dry seeds of wild-type (Col) and each mutant were shaken in ddH$_2$0 or 50 mM EDTA treatments for 30 mins before staining. **(B)** Quantification of three classes of mucilage extrusion levels in the wild type and TF mutants following ddH$_2$0 or 50 mM EDTA treatments. The data was calculated from three independent experiments and are shown as percentages ±SD. The total values show the total number of seeds examined. n.d., not detected. **(C)** Seed colour phenotypes of three biological replicates of wild-type (Col), *myb5, myb23* and *tt2* mutants in single, double and triple mutant combinations and seed colour phenotypes of three biological replicates of wild-type (Col), *gl2, hdg2* and *ttg2* mutants in single, double and triple mutant combinations. Scale bars: 100 µm (A) and 500 µm **(C).**

GH10

TAACGGCTA
TAACCTCTT
TAACTATTA
TAACTGGAT

GH10

TGTAGTTA/TAACTACA
GTTAGTTCTTA/TAAGAACTAAC
ACATAGTTA/TAACTATGT
TAACAGAGT
TTTCAGTTT/AAACTGAAA

PMEI(At1g09370)

TAACAAATT
TAACTTACA
TCAACTCAAA
TAAACATTGA
TAAACAAAAT
TTGTAGTTTG/CAAACTACAA
TGAATGTTA/TAACATTCA

PAE(At1g09550)

TTAGTGTTA/TAACACTAA
CAACAAATC
TAAACGGTAA
AATTGGTTA/TAACCAATT
TTTTGTTTCGA/TCGAAACAAAA

PMEI14

CAACATTAA
TAACAAACC
TCAAGGTTTG/CAAACCTTGA
TTTTGTTTTG/CAAAACAAAAA

PMEI14

TAAACTAACT
ACAAGTTTGTGA/ACACAAACTTGT
TGCAAATGTTTCA/AGAAACATTTG
TTGACGTTTG/CAAACGTCAA
TTTAGGTTTT/AAAACCTAAA
AGTTTGTTTA/TAAACAAACT
TTCAAGGTTTG/CAAACCTTGA

MUM4

TAACCTTCT
CATGAGTTTA/TAAACTCATG
AAAACAAAAC
AAAACAAGAA
TTTATGTTG/CAACATAAA

MUM2 promoter

CAAAACCTCAA
CAACTTTTT
TAACTTTTC
TAACCTATT
AAACATATA
CAAAACAAGAA
TGTTGTTTTGAA/TTCAAAACAACA
GATATGTTGA/TCAACATATC
TTAAGGTTTT/AAAACCTTAA
CCTAGGTTTCA/TGAAACCTAGG
TTCCAGTTGCAAT/ATTGCAACTGGAA

MUM2 promoter

TAACACATC
AAACTTTTC
TAAACGCCTA
TAAAGTTTTTT/AAAAAACTTTA
ATTTGTTTA/TAAACAAAA

MUM2 first intron

TAAACTAGAT
TAACATTTC
CAAACCTCTT
TAACCCAAA
CAAACATTT
TGAACCTTT
TAAACATTTT
TAAACTTGTG
TTATGGTTG/CAACCATAA
TAAATGTTT/AAACATTTA

MUM2 first intron

TAAACTTGTG
CAACTATTG
AAACTATAC
AAAACTATGT
TAACGAATT
TAAACTTCTA
AATTCTGTTT/AAACAGAAT
GCAATGTTTT/AAACATTGG

MUM2 first intron

AAACGAAAT
CAACATCTT
AAACAGTCA
AGATTTGTTA/TAACAAATC
TGATCTGTTA/TAACAGATC

MUM2 first intron

TAAACATTAC
TAAAACTAAAT
GATCCGTTG/CAACGGATC
AATTAGTTA/TAACTAATT
GACATCGTTT/AAACGATGT

MYB5

TAACTGTAA
TTTGGGTTA/TAACCCAAA
AACAAAAGTTTT/AAAACTTTTG
TTAGGGTTTT/AAAACCCTAA

MYB5

TAACTAGAA
CAACTAACC
TAACCTATG
TAACATTAG
AAACTTTGA
AAAACAACCA
ATGATTGTTA/TAACAATCA

TT8

CCAACCATTC
CAACTTTTA
CAACATGTT

TT8

TAACCAACC
TAACCAATC
TAAACACAAA
AAATGAGTTA/TAACTCATT
TTTTCTGTTA/TAACAGAAA
CAGTATTGTTTA/TAAACAATAC

TT8

TAACTCAAA
CAACAACTA
CAACTAATT
CAAAACACTTT

GATL10

AAAACAATGA
AAAACGAGTT
TAACGAGTTTA/TAAAACTCGTTA
TTTATGGGTTT/AAACCCATA
ATGTTTGTTA/TAACAAACA
TTCAATGTTTG/CAAACATTGA

GATL10

TAAACCTAAC
TAACATTTA
TAAACAAAAA
AAAACATTTA
AAACTTTCC
AAACTATAA
AAACTCAAA
TAAACCGTAA
TAACGAGTTA/TAACTCGTTA
ATATCGTTTA/TAAACGATAT
AAATAGGTTA/TAACCAATT
GAGTTAGTTA/TAACTAACT

BGLU44

TAACTTAAG
CAACGCTTA
CAACTAATT
GTTGATGTTA/TAACATCAA
GGCTCAGTTT/AAACTGAGC

BGLU44

AATACGTTTT/AAAACGTATT
AAGAGTTG/CAACTCTT
TTTAGTTT/AAACTAAA
CGTACCGTTTA/TAAACGGTAC

Supplemental Figure 8. Alignment of putative MYB5 binding cis-elements identified in the ChIP-enriched fragments.

The cis-elements containing the core MYB recognition sequence (AAC) are enriched in the ChIP-enriched fragments. Each ChIP-enriched fragment is represented by the corresponding gene names. The MYB5 binding consensus sequence is (T/A)AACN(G/A/T)(T/A)(T/A).

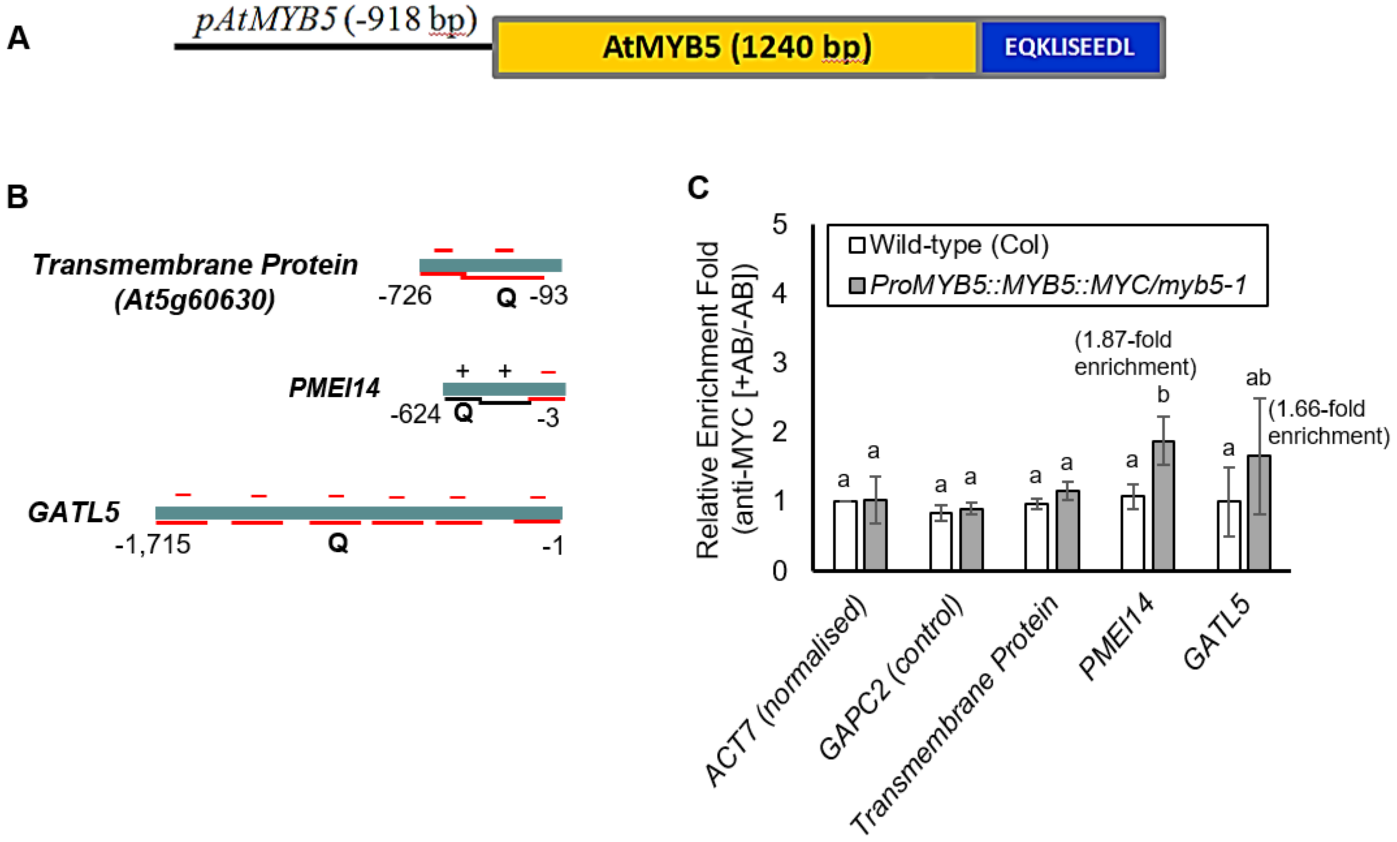

Supplemental Figure 9. ChIP-qPCR analysis of the *Transmembrane Protein (At5g60630), PMEI14 (At1g56100)* and *GATL5 (At1g02720)* promoter regions.

(A) Schematic diagram of *ProMYB5::MYB5::MYC* construct used for ChIP analysis in this study and previously described by Li *et al.* (2020). **(B)** The *Transmembrane Protein (At5g60630)*, *PMEI14 (At1g56100)* and *GATL5 (At1g02720) promoter* regions were investigated using ChIP. Black underlines (and +) represent enriched regions while red underlines (and -) represent regions tested that were not enriched. Numbers represent nucleotide distances towards or past the ATG start codon. Q: Quantified amplicon. (C) MYB5 ChIP-qPCR enrichment values presented as mean fold-enrichment (+AB/-AB, n=3 biological replicates) following normalisation using a control sequence from the Arabidopsis *ACTIN7* gene *(At5g09810).* Enrichment values above the threshold of 2-fold were considered as positively enriched post-normalisation. A sequence from the *GAPC2* gene *(At1g13440)* was used as a non-enriched negative control. Statistical analysis was performed using Statistical analysis (n=3) was performed using one-way ANOVA and Tukey post-hoc test. Bars with different letters are significantly different at $P < 0.05$. Data are shown as mean ± SD.

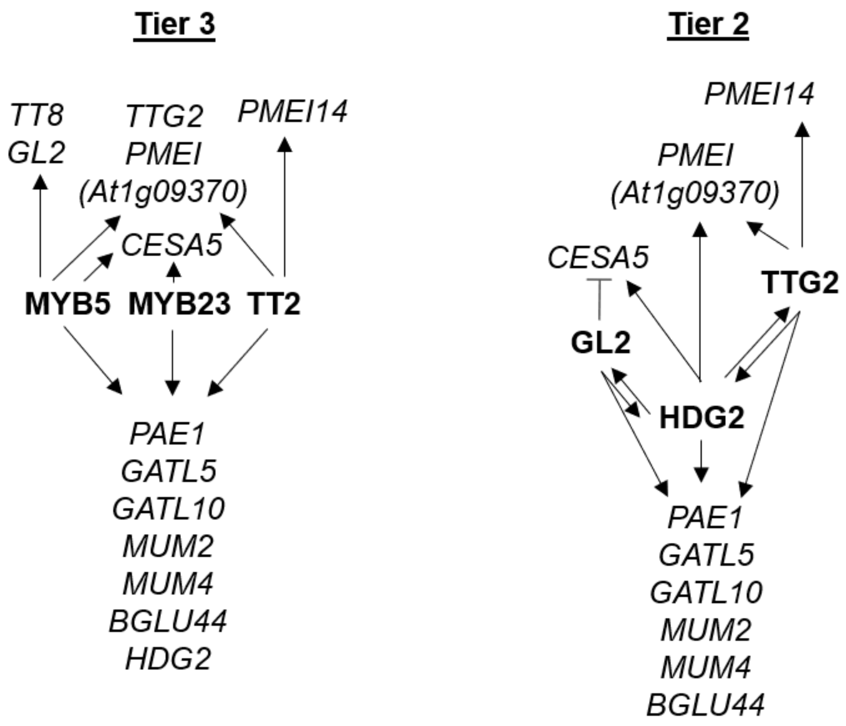

Supplemental Figure 10. Regulation of MYB5 target genes in developing seeds by tier 3 and tier 2 transcription factors.

Two models are presented. All genes are positively regulated by MYB5, MYB23 and TT2 except *PMEI14* and *PMEI (At1g09370)* which are positively regulated by TT2 and TTG2, while MYB5 appears to have mild repressive effect on *PMEI1 (At1g09370)* expression. All genes are regulated by GL2, HDG2 and TTG2 expect the two *PMEI* genes as shown. *HDG2* expression is also partly regulated by GL2 and TTG2 indicating a feedback mechanism within the tier 2 of the seed coat regulatory pathway.

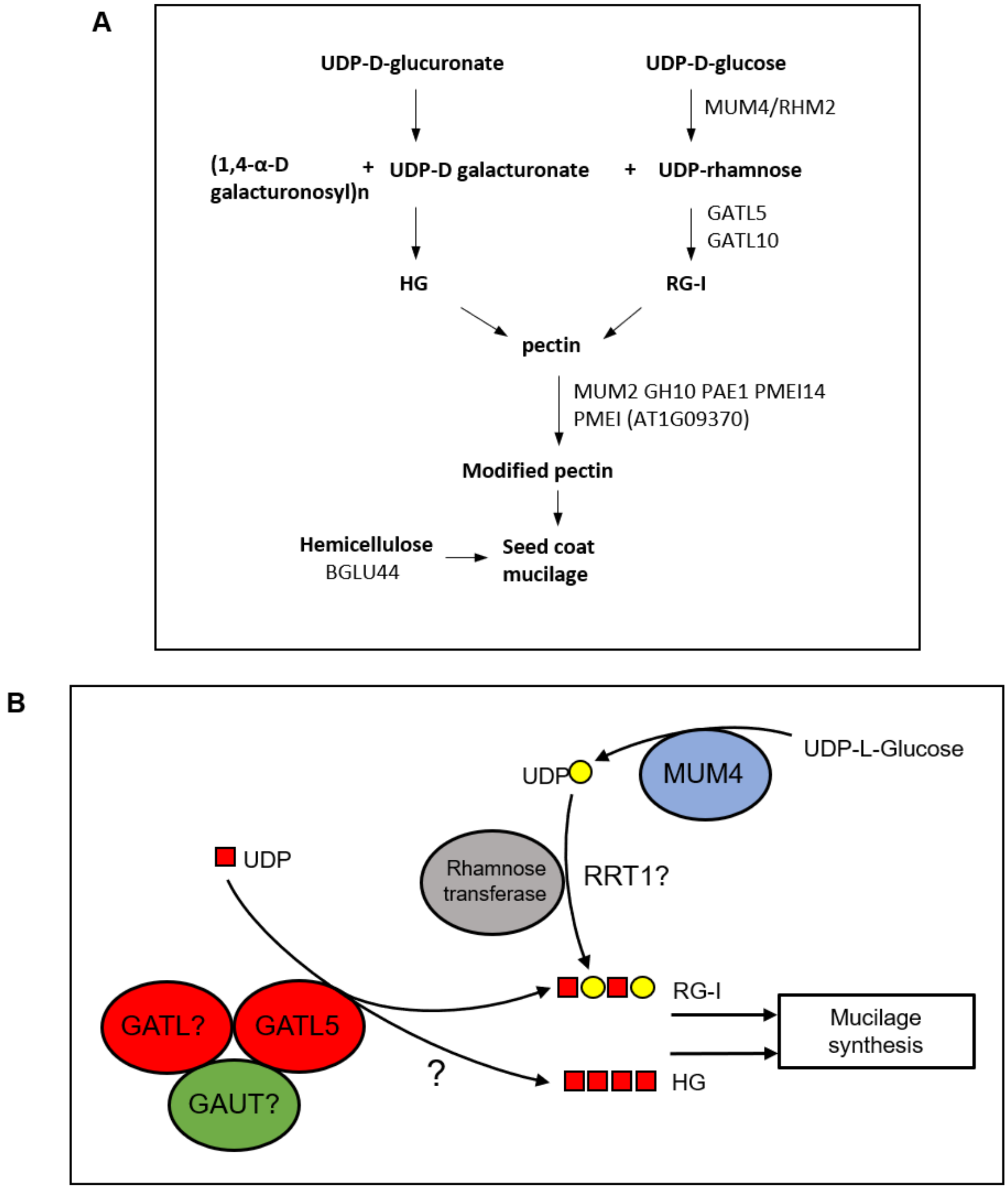

Supplemental Figure 11. A simplified TTG1-regulated seed coat mucilage biosynthesis pathway.

(**A)** Mucilage pathway genes known to participate in the TTG1-regulated mucilage pathway examined in this study are shown (adapted from Li *et al.,* 2020). RG-I Rhamnogalacturonan-I; HG, homogalacturonan. **(B)** Model for AtGATL5 function in pectin synthesis. GATL5 may collaborate with at least one lumen bound GAUT protein(s) in a heterotrimeric core complex similar to the GAUT1:GAUT7 core complex in HG pectin synthesis. GATL5 may catalyse the addition of GalA residues (red square blocks) onto both RG-I and HG pectin chains while UDP-L-rhamnose sugars (yellow circles) converted from UDP-D-glucose by MUM4 are added by at least one transferase enzyme(s). (Adapted from Western, 2006; Oka *et al.,* 2007, Kong *et al.,* 2011, 2013; Takenaka *et al.,* 2018).

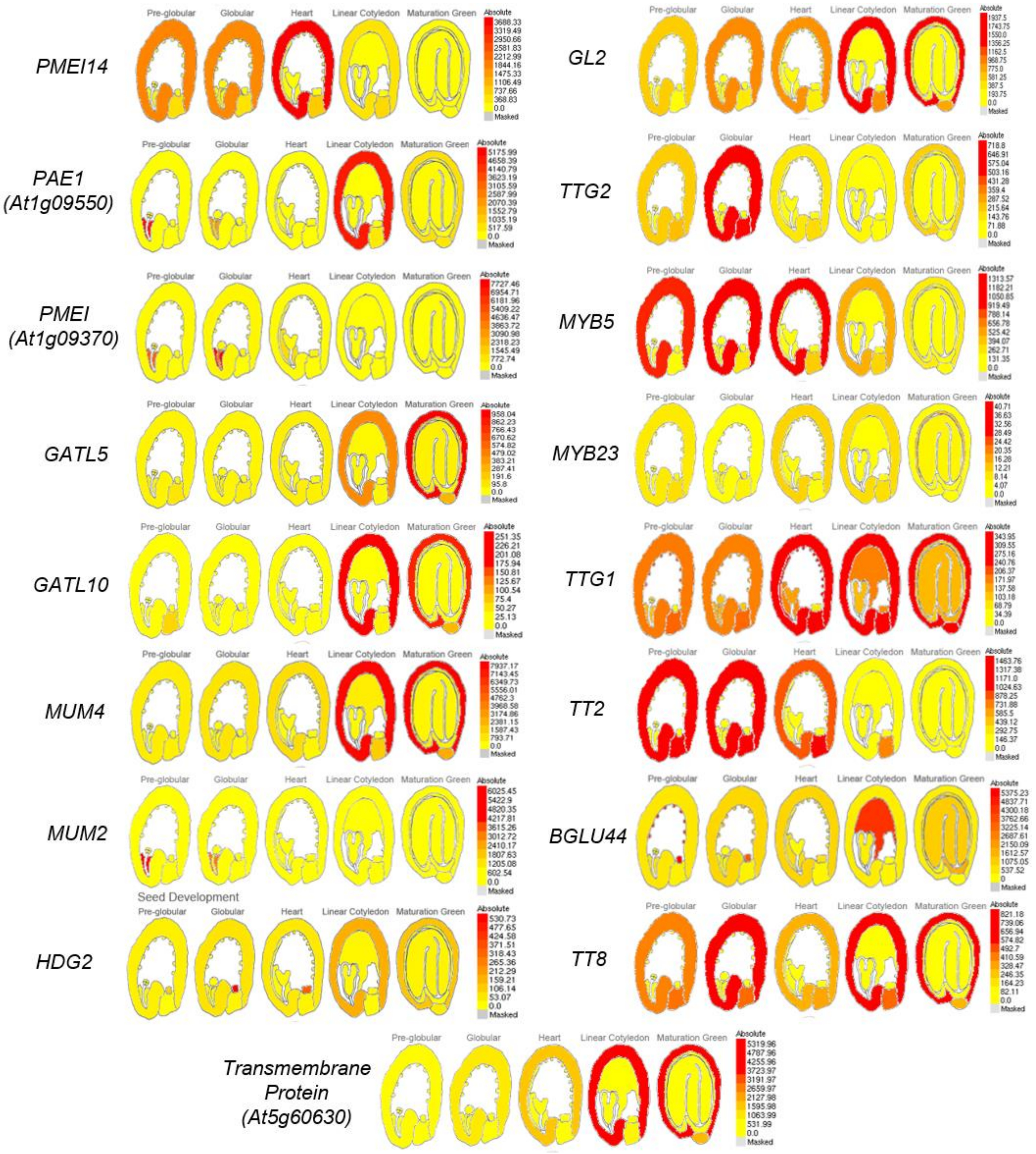

Supplemental Figure 12. Spatial and temporal expression patterns throughout seed development for genes listed in this study as indicated by the Arabidopsis eFP-Browser database.

Most MYB5 target genes are co-expressed during the later stages of seed development while *PMEI14, GL2* and *TTG2* are expressed in younger seeds. *TTG1* and *TT8* are expressed during all developmental stages. (Figure adapted from the Arabidopsis eFP Browser Database, University of Toronto, cited as Winter *et al.,* 2007; Bassel *et al.,* 2008; Le *et al.,* 2010).

Supplemental Table 1. Summary of *PMEI14* T-DNA insertion mutants. *The SM_3_38019 insertion occurs in both an intron of the *PMEI14.1* and an exon of the *PMEI14.2* splice variants.

T-DNA insertion	Location	Gene primers	T-DNA primer	Mucilage
**SAIL_561_A03**	Promoter	1g561F and 1g561R	LbB1.3	Yes
**SM_3_38019**	Intron/exon*	730(1) and 730(4)	730(2)	Yes
**FLAG_367F01**	Intron	561 qRT beta F1 and 561 qRT beta R1	LB4	Yes
**GK_384A06**	Intron	(as above)	o8409	Yes
**SALK_113576C**	Intron	(as above)	LbB1.3	Yes

Supplemental Table 2. A subset of genes with differential expression in *ttg1-1* vs. wild-type (L*er*) transcriptome analysis (Li *et al.,* 2020) and predicted MYB-DNA binding motifs present in the promoter of each gene. Mucilage from mutant seeds is present at wild-type levels (+) or modified levels (-).

Gene Locus ID	Name or Annotation	Predicted MYB Binding Motifs	Mucilage	Fold Change	P-value	Up/ Down
**At1g56100**	Pectin methylesterase inhibitor/PMEI14	MYBPLANT x2, MYBCORE x3, MYBPZM x1, MYBGAHV x2, MYB1LEPR x1, MYBST1 x1	+	-200.25	1.49E-04	Down
**At1g09370**	Pectin methylesterase inhibitor/PMEI	MYBPLANT x1, MYBCORE x1, MYBST1 x, MYBGAHV x1, MYB1LEPR x1	+	-3.84	2.85E-02	Down
**At1g09550**	Pectin acetylesterase/PAE	MYB1AT x1, MYBCOREEATCYCB1 x1, MYBPLANT x3	+	-18.21	1.19E-03	Down
**At4g33810**	Glycosyl hydrolase/GH10	MYBPLANT x4, MYB1AT x1, MYBPZM x1, MYBCORE x3	+	-2.74	1.79E-02	Down
**At3g18080**	Beta-glucosidase44/BGLU44	MYBST1 x5, MYBPLANT x1, MYBPZM x1, MYBCOREEATCYCB1 x3, MYB1AT x1, MYBGAHV x1	+	-2.40	3.76E-02	Down
**At1g02720**	Galacturonosyl transferase-like 5/GATL5	MYBPLANT x2, MYBPZM x1, MYBST1 x3, MYBCORE x4, MYB1AT x6, MYB26PS x1, MYBGAHV x1, MYBCONSENSUSAT x2, MYBATRD22 x3, MYBCOREATCYCB1 x2	-	-7.74	1.79E-03	Down
**At3g28340**	Galacturonosyl transferase-like 10/GATL10	MYBST1 x2, MYB1LEPR x1, MYBGAHV x1, MYBPLANT x3, MYBCORE x2, MYBCOREEATCYCB1 x1	+	-3.80	5.38E-03	Down
**At5g63800**	Beta-galactosidase/MUM2 *(Promoter Region)* *(First Intron Region*ɸ*)*	MYBPZM x2, MYBPLANT x3, MYBST1 x1, MYB1AT x1, MYBCORE x5, (MYBST1 x4ɸ, MYBPZM x1ɸ, MYB1AT x1ɸ, MYBGAHV x3ɸ, MYBCORE x4ɸ)	-	-2.63	1.01E-02	Down
**At1g53500**	Rhamnose synthase/MUM4	MYB1AT x7, MYBPLANT x1, MYBCORE x4, MYBCOREEATCYCB1 x1, MYBGAHV x1, MYB-related x1	-	-3.10	1.19E-03	Down
**At3g13540**	MYB5	MYBCORE x1, MYBCONSENSUS x1, MYB2AT x1, MYBATRD22 X1, MYBPLANT x1, MYBPZM x1, MYB1AT x3, MYB1LEPR x1, MYBGAHV x1	-	NC	NC	NC
**At4g09820**	TRANSPARENT TESTA8 (TT8)	MYB1AT x7, MYBPLANT x2, MYBCORE x5, MYBPZM x2	-	-13.99	6.08E-05	Down

Predicted MYB binding motifs present in the promoters (~1,000 nucleotides upstream of ATG) were obtained using cis-PLACE and AtCIS database analysis (MYB1AT, A/TAACCA, MYB2AT, TAACTG, MYBCORE, CNGTTA/G, MYBST1, GGATA, MYBGAHV, TAACAA, MYB1LEPR, GTTAGTT, MYBCOREEATCYCB1, AACGG, MYBATRD22, CTAACCA, MYBPZM, CCA/TACC, MYBPLANT, A/CACCA/TA/CC, MYB26PS, GTTAGGTT, MYBCONSENSUSAT, C/TAACG/TG). ɸThe *MUM2* first intron region contains MYBST1, MYBPZM, MYB1AT, MYBGAHV and MYBCORE motifs. NC: no change. Roles for *GATL5, MUM2, MUM4, GL2, TT8* and *TTG2* in mucilage pathways have previously been reported (Rerie *et al.,* 1994; Johnson *et al.,* 2002; Western *et al.,* 2004; Usadel *et al.,* 2004; Baudry *et al.,* 2006; Dean *et al.,* 2007; Kong *et al.,* 2013) and mutant seeds for each of these genes display a reduced or modified mucilage phenotype. Data presented as a subset of genes published by Li *et al.* (2020).

Supplemental Table 3. Summary of enriched promoter and intron regions across 6 biological replicates using ChIP-PCR and ChIP-qPCR screening. *ACTIN7* and *GAPC2* were used as negative control sequences and were not enriched.

**MYB5 ChIP Positive Genes**		**ChIP Biological Replicates**					
**Gene Name**	**Locus ID**	**1**	**2**	**3**	**4**	**5**	**6**
PMEI14	At1g56100	+	+	+	+	+	+
PMEI	At1g09370	NT	NT	+	+	+	+
PAE	At1g09550	+	+	+	+	+	+
GATL10	At3g28340	NT	+	NT	+	+	+
***MUM2* (promoter) Φ**	At5g63800	NT	NT	+	+	+	+
***MUM2* (first intron) Φ**		NT	+	NT	+	+	+
MUM4	At1g53500	NT	+	NT	+	+	+
MYB5	At3g13540	+	+	+	+	+	+
TT8	At4g09820	NT	+	NT	+	+	+
GH10	At4g33810	NT	+	+	+	+	+
BGLU44	At3g18080	NT	NT	NT	+	+	+
GATL5	At1g02720	NT	-	NT	-	-	-
Transmembrane Protein	At5g60630	NT	-	NT	-	-	-
***ACTIN7* (normalisation control)**	At5g09810	-	-	-	-	-	-
***GAPC2* (negative control)**	At1g13440	-	-	-	-	-	-

NT: Not tested, (-): no enrichment in PCR, (+): PCR positive enrichment post normalization. ΦThe *MUM2* promoter and first intron region are both enriched in our MYB5 ChIP analysis.

Supplemental Table 4. Amplicon sizes for candidate ChIP-positive and ChIP-negative promoter and intron regions.

Gene Name	Locus ID	Enriched Regions	Non-enriched Regions
PMEI14	At1g56100	-465 to -208<br>-624 to -445	-215 to -3
PMEI	At1g09370	-318 to -30	-992 to -657<br>-605 to -294
PAE	At1g09550	-1,046 to -735	-349 to -35
GATL10	At3g28340	-347 to -44<br>-807 to -526	-550 to -331<br>-1,190 to -904
***MUM2* (promoter) Φ**	At5g63800	-804 to -531<br>-313 to -14	-521 to -291
***MUM2* (first intron) Φ**		+171 to +350<br>+325 to +658<br>+1,346 to +1,515<br>+1,495 to +1,708	+635 to +976<br>+950 to +1,367<br>+1,687 to +1,929
MUM4	At1g53500	-503 to -235	-741 to -481
MYB5	At3g13540	-906 to -632<br>-384 to -27	-661 to -361
TT8	At4g09820	-306 to -8<br>-1,309 to -1,072<br>-1,697 to -1,319	-710 to -302<br>-1,029 to -698
GH10	At4g33810	-907 to -622<br>-547 to -169	-1,383 to -1,103<br>-1,127 to -930
BGLU44	At3g18080	-1,239 to -835<br>-1,635 to -1,303	-780 to -422<br>-413 to +3
GATL5	At1g02720	None	-1,715 to -1,580<br>-1,403 to -1,191<br>-1,084 to -852<br>-803 to -633<br>-626 to -425<br>-147 to -1
Transmembrane Protein	At5g60630	None	-726 to -492<br>-519 to -93

Amplicon size numbers are relative to the ATG start codon for each gene promoter. ΦThe *MUM2* promoter and first intron region are both enriched in our MYB5 ChIP analysis.

Supplemental Table 5. List of primer oligonucleotide sequences used in this study presented in 5' to 3' orientation.

Primers used for PCR and cloning during plasmid construction:

1g561F	CACCGATGGAGACTGCAGAGGAGAACG
1g561R	GCGAAGTATATTTCCTATGATTG
PMEI14 COMP F1	CACCTTATGACAAAGGTGTTGCCCC
PMEI14 COMP R1	AGCATACTCTATCGATTTGGCGGCC
PMEI14 COMP R3	CCTTCTTTCGTTCCTCCTCCCAGTGAC
PMEI14 PRO COMP R1	TATGCGAAGTATATTTCCTATGATTGATTATG
PMEI14.1 OVERLAP F1	CAATCATAGGAAATATACTTCGCATAATGACGATA ATGATCAAGTTTCTCC
PMEI14.1 OVERLAP R1	CTATGCCTCAGATGAAGTTACATCGAC
1g955F	CACCGCCTCATCTAATACGACGCGTAATG
1g955R	GAGTTGTCAGTATATACGCAGCC

Primers used for qRT-PCR analysis of *PMEI14* mRNA splice variants:

PMEI14 START F1	ATGACGATAATGATCAAGTTTCTCCTG
PMEI14 STOP R1	AGCATACTCTATCGATTTGGCGGC
PMEI14 STOP R2	CTATGCCTCAGATGAAGTTACATCGAC
UBQF	TCCGGATCAGCAGAGGCTTA
UBQR	TCAGAACTCTCCACCTCAAG

Primers used for PCR screening of T-DNA insertion mutant lines:

730(1)	AAGAGCGCTGAATAGAAAGGG
730(3)	GGCACCTAAACTACTGTCGGAAGCC
730(4)	AGCGATCAATGATTCTCATGG
o8409	ATATTGACCATCATACTCATTGC
LB4	CGTGTGCCAGGTGCCCACGGAATAGT
GATL5 KONG F	AACTCGAAATTGGCATCAATG
GATL5 KONG R	ATTCTCTGCTTTCTTCTCCGC
MUM4 F1	TTCTCATTACTGGAGCTGCTGG
MUM4 R1	TGCAACCGGTGGCGAAGTTCA
LbB1.3	ATTTTGCCGATTTCGGAAC

Primers used for qRT-PCR gene expression analysis:

PMEI14 EST F1	GCAATGTCTTGAATCCGACCCAACCTCCG
PMEI14 EST R1	CTCACTTCTTTCGTTCCTCCTCCCAGTGAC
MUM4 qRT F2	GGTTCGGGTATAGGTTTCAAGG
MUM4 qRT R2	GTTTAGGTCTGAGGAGATTGGC
MUM2 qRT F1	CCCTGCTGGACAACCTTCT
MUM2 qRT R1	CCAAGAGGATCACCACCTTC
GATL5 3UTR F1	GGTTTTCAACTGGAAACAACTATGTTG
GATL5 3UTR R1	ACGGTCTGGTTGAACTGTCC

Supplemental Table 5 (continued)

809 qRT F2	TGAACGTGACAGTAAGCGAAG
809 qRT R2	CGAGTTCACAATGGCGTAAG
HDG2 qRT F1	TGCTTCGTGTCCTAATTGTGGTGGTCC
HDG2 qRT R1	TGCCTACGTATTTAGCTGCGATTGCGG
955 qRT F3	GCGTTGAAGACCTTTTCGAAG
955 qRT R3	CCAATCTCCAACAGCCACAG
BGLU44 qRT F1	GCTCAAGGACTACATGACACC
BGLU44qRT R1	GTAATCAACATAAACGATCCCAAAC
GL2 qRT F	TCGCTGTGGTTACCTGTTTCTCCAGCTCTTCTC
GL2 qRT R	GCCTCTGTCTTGTCCCTTGGATAAGTTTGC
TTG2 qRT F	GTAGCCAATGTGATGAAGGAGAGC
TTG2 qRT R	GTCCATACTTTCTCCACCTGAATCC
CESA5-qPCR-F	CGAAGAGGGCACCAAAGGCACTAATG
CESA5-qPCR-R	GCCCACCATTCTCCATACCAGCAG
TT8 qRT F	CTCCATCTGGGATGCCAGG
TT8 qRT R	CCACTTAGCCATACGTGCTTCC
UBQF	TCCGGATCAGCAGAGGCTTA
UBQR	TCAGAACTCTCCACCTCAAG

Primers used for ChIP-qPCR analysis:

PMEI14 CHIP F1	GGTCTGGAAACGATTCAAGGTTTGGC
PMEI14 CHIP R1	CGCGAGCTGCTCTTCTTCTATTACTCC
PMEI14 CHIP F2	GCTCGCGTTTGAATGGTCAG
PMEI14 CHIP R2	GCGAAGTATATTTCCTATGATTGATTATG
PMEI14 CHIP F3	GAGACTGATGATGGTCACAGC
PMEI14 CHIP R3	CCTTGAATCGTTTCCAGACC
937 CHIP F1	GGATAGCTCCTCAAACATCTTTC
937 CHIP R1	CACATGAGAGATTGTCACCTAGA
937 CHIP F2	GCGATTGAAGACACACACAC
937 CHIP R2	GTCAAGGCCACAATTACACTCAAC
937 CHIP F3	GTTGAGTGTAATTGTGGCCTTGAC
937 CHIP R3	GTAAATTGAGTATGTGGTCATTTGAG
955 CHIP F1	CTTTTTAGTGTTAAAAGCCTCATC
955 CHIP R1	TGCAAGAGTTCAAAAAGCTAC
955 CHIP F3	CGTAATGGAGGTTTAGGCCCC
955 CHIP R3	GGAGACGTACTAGAGTTGTCAG
MUM4 CHIP F3	CCACGATTAACCTTCTAAAATAAGG
MUM4 CHIP R3	CGGATTTCTCGTGAGTCTAGC
MUM4 CHIP F4	CGCTAGACTCACGAGAAATCC
MUM4 CHIP R4	GAGTCAGATAACCCAAGAGAG
GATL5 CHIP F5	GTGCTAATTACTTTGGTTTGAC
GATL5 CHIP R5	GGGTTACAAGTTCGCACAAC
GATL5 CHIP F6	CTACTAAAATATTTCTAACCATGC
GATL5 CHIP R6	CGTACCATATGAACCATGAACAG
GATL5 CHIP F7	GCGGGACCTCATTTCCAAG
GATL5 CHIP R7	GGGGGGTCGAATAATGCTTTC

Supplemental Table 5 (continued)

GATL5 CHIP F8	CTATTATATAGGGTATCATGTTAGATC
GATL5 CHIP R8	CTTTTTATGGCGCATGTTTAAGCAG
GATL5 CHIP F9	AACGTGGTTATCTTTTTTAGAGCAC
GATL5 CHIP R9	GATCAATTTATGTATTGTGAAGAGC
GATL5 CHIP F10	CGGCGCAGAGTAGCATTGAC
GATL5 CHIP R10	GCGAGGAATGCAGAAGAAACTACG
GH10 CHIP F1	TCACTAAAGATGCGGGATTCG
GH10 CHIP R1	GAATGCACAAAACACCAGAGG
GH10 CHIP F2	GTGAGATTATCATACTCAAATAAGACTTG
GH10 CHIP R2	CGTATTCCAACCACACCCAC
GH10 CHIP F3	CTTTCGTATTCCAACCACACCC
GH10 CHIP R3	CGCTAGCCCAGGGACCATTTG
GH10 CHIP F4	GGCACGCGAGGGTTATGCTC
GH10 CHIP R4	GTATGTCTCGACACTATGATG
MYB5 CHIP F1	CTTCTTCCTCCGTCAGCAGAAC
MYB5 CHIP R1	CCTCTCCTTTATTACAGTTAGTCAATTTC
MYB5 CHIP F2	GAAATTGACTAACTGTAATAAAGGAGAGG
MYB5 CHIP R2	CATCCGATTATACCAGCTCCTAC
MYB5 CHIP F3	GTAGGAGCTGGTATAATCGGATG
MYB5 CHIP R3	TCTTTCCACTAGGGTTTCGTG
TT8 CHIP F1	TGGTGAACCAACCATTCAAAAATC
TT8 CHIP R1	GAGATACGAAAACGTGGTAGC
TT8 CHIP F2	CCGGTCAGGTCAACAATTCTAC
TT8 CHIP R2	ACCAGACGTTGGATGAGAAAG
TT8 CHIP F3	CACAGAAGCAGAAGCAAAAGCC
TT8 CHIP R3	TGACCTGACCGGTAAGTAAAG
TT8 CHIP F4	ATTTGTGCCTAACCAACCTCTC
TT8 CHIP R4	ATTATGTAACTCTGACGACGGGC
TT8 CHIP F5	CATGGGATTACGGTCCAACTG
TT8 CHIP R5	ACCCAAGTTCTTCCATTCCAGA
MUM2 CHIP F4	CCAAGTTCCAGTTGCAATGTCG
MUM2 CHIP R4	TTCAAAACAACAAAATGGTCC
MUM2 CHIP F5	CTTGCTCATTTTTAGCATTGACTTG
MUM2 CHIP R5	TGCTTTTCACCTTTGTCTTACG
MUM2 CHIP F6	CGTAAGACAAAGGTGAAAAGC
MUM2 CHIP R6	CTCTTCCTCTCTGATGTTTCTTTTACTG
MUM2 INT F1	CGTGATAAACTTCTATTTTCGCC
MUM2 INT R1	CACAAATCACTTAAAATGTTTGATTG
MUM2 INT F2	CAATCAAACATTTTAAGTGATTTGTG
MUM2 INT R2	CGTAATGCATGTCTTTTTCGT
MUM2 INT F3	ACGAAAAAGACATGCATTACG
MUM2 INT R3	CGTGTTGAGACCCGTGACTG
MUM2 INT F4	CAGTCACGGGTCTCAACACG
MUM2 INT R4	AAATGGAAGCACAGACAACGG
MUM2 INT F5	CAGATCCGTTGTCTGTGCTTCC
MUM2 INT R5	ACGTGATTGTGTTAGAGTTGTC
MUM2 INT F6	ACACCCGAGGTAAATGTTTCCA

Supplemental Table 5 (continued)

MUM2 INT R6	TATGACACAAGTTTATGGTCCAACC
MUM2 INT F7	GGTTGGACCATAAACTTGTGTCATA
MUM2 INT R7	TATCACGCAACAAAAAGTACATGA
BGLU44 CHIP F1	AGCATCAATATACTTTCTACTTACG
BGLU44 CHIP R1	CATGGTGAGTGAGGTTTGAC
BGLU44 CHIP F2	GGCCGGACACGATAATTCTG
BGLU44 CHIP R2	GGAGATGACACAAAAGGACATC
BGLU44 CHIP F3	AATGCTTGTTAGACTCGACG
BGLU44 CHIP R3	GAACACACAGAAGCCGACAAG
BGLU44 CHIP F4	GGTCAATGTGTCTATGATTTGGG
BGLU44 CHIP R4	GCCCTGTCACATAACATCAAC
EP CHIP F1	TCTCTGAGAAACTATGTGTATATGTGTG
EP CHIP R1	GGGTACCTATGACGAATGGG
EP CHIP F2	GAGTTTGGACTAGATAAAGATGGG
EP CHIP R2	CACACATATACACATAGTTTCTCAGAG
GAPC2 CHIP F2	GGTGCCGCCAAGGTATCATTTC
GAPC2 CHIP R2	AGGACTGAGGGTCCTTGATGG
ACTIN 7 CHIP F1	AGAGATCACCGCTCTTGCACC
ACTIN7 CHIP R1	AGGCGGAATTGGGATTTTTACC
